# Stunting in childhood: an overview of global burden, trends, determinants, and drivers of decline

**DOI:** 10.1093/ajcn/nqaa159

**Published:** 2020-08-29

**Authors:** Tyler Vaivada, Nadia Akseer, Selai Akseer, Ahalya Somaskandan, Marianne Stefopulos, Zulfiqar A Bhutta

**Affiliations:** Centre for Global Child Health, Hospital for Sick Children, Toronto, Canada; Centre for Global Child Health, Hospital for Sick Children, Toronto, Canada; Dalla Lana School of Public Health, University of Toronto, Toronto, Canada; Centre for Global Child Health, Hospital for Sick Children, Toronto, Canada; Centre for Global Child Health, Hospital for Sick Children, Toronto, Canada; Centre for Global Child Health, Hospital for Sick Children, Toronto, Canada; Centre for Global Child Health, Hospital for Sick Children, Toronto, Canada; Dalla Lana School of Public Health, University of Toronto, Toronto, Canada; Center of Excellence in Women and Child Health, the Aga Khan University, Karachi, Pakistan

**Keywords:** child, infant, nutrition, height, length, linear growth, stunting

## Abstract

**Background:**

Progress has been made worldwide in reducing chronic undernutrition and rates of linear growth stunting in children under 5 y of age, although rates still remain high in many regions. Policies, programs, and interventions supporting maternal and child health and nutrition have the potential to improve child growth and development.

**Objective:**

This article synthesizes the available global evidence on the drivers of national declines in stunting prevalence and compares the relative effect of major drivers of stunting decline between countries.

**Methods:**

We conducted a systematic review of published peer-reviewed and gray literature analyzing the relation between changes in key determinants of child linear growth and contemporaneous changes in linear growth outcomes over time.

**Results:**

Among the basic determinants of stunting assessed within regression-decomposition analyses, improvement in asset index score was a consistent and strong driver of improved linear growth outcomes. Increased parental education was also a strong predictor of improved child growth. Of the underlying determinants of stunting, reduced rates of open defecation, improved sanitation infrastructure, and improved access to key maternal health services, including optimal antenatal care and delivery in a health facility or with a skilled birth attendant, all accounted for substantially improved child growth, although the magnitude of variation explained by each differed substantially between countries. At the immediate level, changes in several maternal characteristics predicted modest stunting reductions, including parity, interpregnancy interval, and maternal height.

**Conclusions:**

Unique sets of stunting determinants predicted stunting reduction within countries that have reduced stunting. Several common drivers emerge at the basic, underlying, and immediate levels, including improvements in maternal and paternal education, household socioeconomic status, sanitation conditions, maternal health services access, and family planning. Further data collection and in-depth mixed-methods research are required to strengthen recommendations for those countries where the stunting burden remains unacceptably high.

## Introduction

High rates of chronic malnutrition in young children persist globally, a condition that is strongly linked to poverty. Maternal malnutrition can start the process of linear growth faltering in utero, contributing to intrauterine growth restriction and low birth weight. Suboptimal feeding practices in infancy coupled with a high burden of infectious diseases also predict poor child growth. Linear growth stunting, defined as a height-for-age *z* score (HAZ) ≥2 SDs below the median, is an easily recognizable and quantifiable physical indicator of chronic childhood malnutrition.

Children whose growth is stunted are more likely to experience higher rates of mortality, morbidity, and suboptimal cognitive and motor development (1). Meta-analyses of 5 prospective cohort studies have shown that a unit increase in HAZ for children ≤2 y was associated with a 0.22-SD improvement in cognitive function later in childhood at 5–11 y (2), illustrating the lingering effects of early-life chronic malnutrition. This has serious implications for population health and the fulfilment of the intellectual and economic potential of low- and middle-income countries (LMICs). Despite these associations, stunting has the potential to be misused as a measure of population health, as poor nutritional status can affect the health, growth, and development of children whose linear growth falls above the HAZ cutoff (3). It is helpful to conceptualize stunting as a robust indicator of a deficient environment, which has strong associations with adverse outcomes in the short and long term, rather than the sole cause of poor cognitive development or future risk for chronic diseases (4).

**FIGURE 1:**
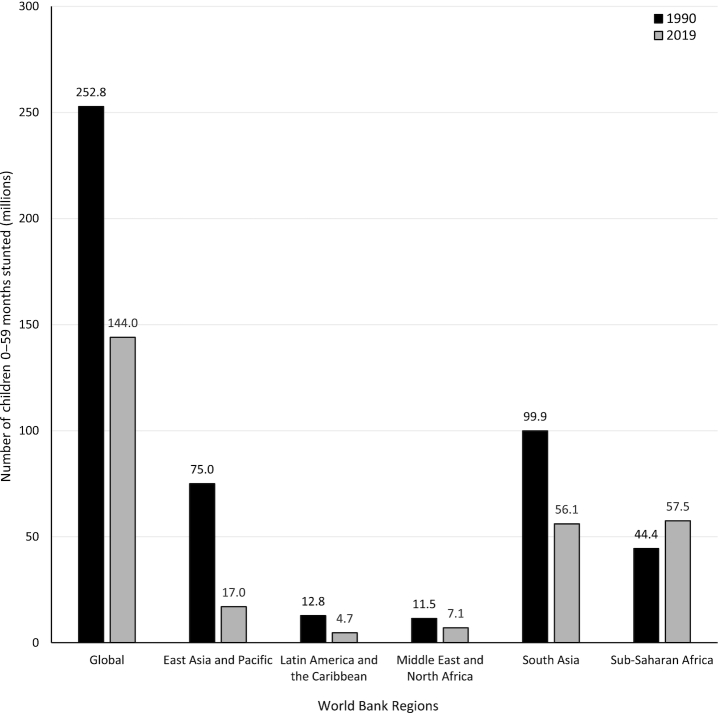
Global and regional comparison of the total number of children aged 0–59 mo experiencing linear growth stunting in 1990 and 2019. Source of data: UNICEF, World Bank Group joint malnutrition estimates, 2020 edition (6). Data not available for Europe and Central Asia.

**FIGURE 2:**
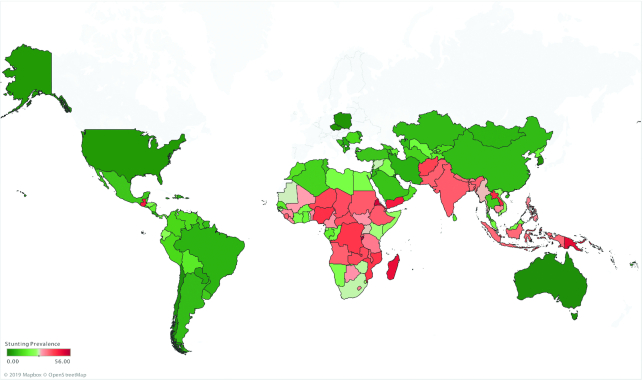
Stunting prevalence for children under 5 y based on the most recently available country-level estimates. Map based on longitude (generated) and latitude (generated). Color shows sum of stunting prevalence. Details are shown for country. Source of data: UNICEF, WHO, World Bank Group joint malnutrition estimates, 2020 edition (6). Data not available for Europe and Central Asia.

**FIGURE 3:**
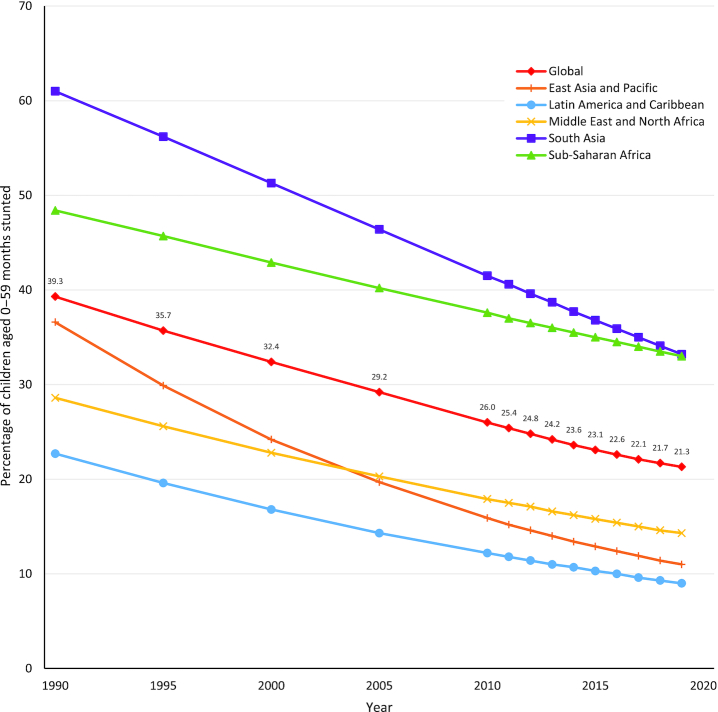
Global and regional trends in stunting prevalence, 1990–2019. Source of data: UNICEF, WHO, World Bank Group joint malnutrition estimates, 2020 edition (6). Data not available for Europe and Central Asia.

**FIGURE 4:**
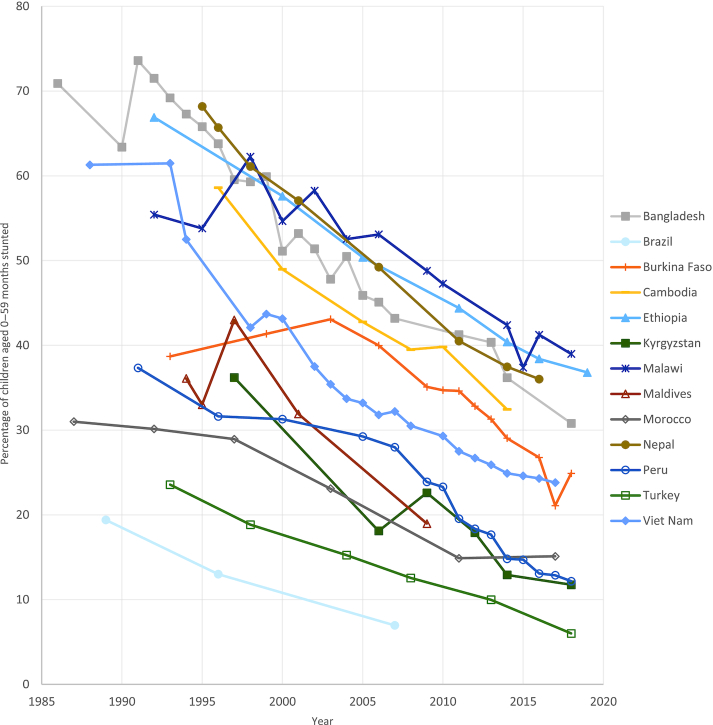
Stunting prevalence, top-performing countries. Source of data: UNICEF, WHO, World Bank Group joint malnutrition estimates, 2020 edition (6).

There has been global progress on reducing rates of child stunting in recent decades, but this progress has been uneven (see **Panel 1**, **Figures 1**–**4**, **Supplemental Figure 1**). Some particularly high-performing countries have reduced stunting prevalence by >30 percentage points in the past 30 y, while others have made negligible progress. It is crucial to examine the key determinants and drivers of stunting reduction so that individual countries can learn what works in order to implement targeted policies and programs. Countries that prioritize the implementation and scale-up of evidence-based, nutrition-sensitive, and nutrition-specific policies and programs stand to make great improvements in human capital development and economic productivity, as these initiatives generally have very high benefit–cost ratios (5). There is also a moral imperative to act, as all children have the right to grow and develop optimally in order to reach their full developmental potential. Targeted and concerted action at the national level will be essential to achieve the Sustainable Development Goals related to child health and nutrition.

As an introduction to this supplement issue, this article includes an overview of the epidemiology of stunting across LMICs (Panel 1, Figures 1–4, Supplemental Figure 1) and a summary of existing conceptual thinking around the major determinants of chronic childhood malnutrition and stunting (**Panel 2**). The main objective of this article is to synthesize the available global evidence on the drivers of national declines in stunting prevalence and compare the relative effect of major drivers of stunting decline between LMICs. The remainder of this article focuses on this objective. Specifically, we sought to synthesize the global evidence examining drivers of reductions in child stunting over time. To this end, we conducted a systematic review of published peer-reviewed and gray literature that analyzed the relation between changes in key determinants of child growth and contemporaneous changes in growth outcomes over time. These theoretical determinants, described in Panel 2 and **Supplemental Figure 2**, included contextual factors, interventions, policies, strategies, programs, and other initiatives that may have accounted for reductions in under-5 child stunting prevalence over time in LMICs.

Panel 1: **Child Stunting Epidemiology**

**The changing global burden of childhood linear growth stunting**

Although stunting rates have been decreasing over the past several decades, an estimated 21.3% (144 million) of children under 5 y of age globally experienced stunted growth in 2019 (6). Both regional and within-country disparities exist, with prevalences ranging from 34.5% in eastern Africa to 4.5% in eastern Asia as of 2019 (6). Globally, there were ∼109 million fewer children experiencing stunting in 2019 compared with 1990. However, despite making modest progress in reducing prevalence, due to substantial population growth the total number of children experiencing stunting in the African region has increased by ∼13.1 million since 1990 (see Figure 1). An estimated 17% of mortality burden in children under 5 y is associated with stunting (1). Compared with children with HAZ > −1, children with HAZ between −2 and −3 have a 118% (HR: 2.18) and 138% (HR: 2.38) higher risk of dying from pneumonia or diarrhea, respectively (7). Those children who are severely stunted (HAZ ≤ −3) are at even higher risk (pneumonia mortality—HR: 6.39; diarrhea mortality—HR: 6.33) (7).

Those countries with the highest levels of stunting prevalence are concentrated in South and Southeast Asia and sub-Saharan Africa, as depicted on the map in Figure 2. A chart of the most recent country-level estimates of stunting prevalence worldwide can be found in Supplemental Figure 1. While all global regions have experienced decreases in stunting prevalence since 1990, this progress has been uneven. The regions of South Asia and East Asia and the Pacific have seen the greatest improvements, reducing stunting prevalence by ∼25 percentage points over the past 30 y (see Figure 3).

**National trends in child stunting in top-performing countries**

Those countries that have achieved substantial reductions in stunting prevalence over the past ∼30 y are geographically dispersed among several regions worldwide. Figure 4 depicts the trends in stunting prevalence in a sample of 13 of the best-performing countries globally, which were selected based on consultations with experts. Although the baseline prevalence and rate of reduction in stunting vary for each of these countries throughout the period examined, one consistent pattern emerges that characterizes several of these top performers: an initial period of stagnation followed by a consistent decline.

For example, between 1988 and 1993 Vietnam initially experienced stagnation (∼61%) but saw a very steep decline between 1993 and 1998, followed by relatively consistent reductions until 2015 (∼25%). In Burkina Faso, this initial plateau lasted until 2006, after which dramatic and consistent reductions were seen. Although data are not available for Nepal before 1995, since then, Nepal (68.2%) and Bangladesh (65.8%) followed a very similar and consistent pattern of decline until 2014, reducing stunting prevalence by ∼30 percentage points. An examination of the contributing factors to Peru's own steep decline between 2008 and 2016 (28.2–13.1%) is the subject of an in-depth case study within this supplement issue.

It is important to note that there exists considerable variation in both stunting burden and trends within countries. This subnational variation is closely related to socioeconomic and geographic disparities, including indicators such as parental education, household wealth, and rural location. These subnational inequities are also analyzed and discussed in detail within each of the case-study articles within this supplement issue.

Panel 2: **Determinants of Linear Growth in Childhood**

**Theoretical determinants of linear growth faltering in young children**

Policies and programs designed to alleviate childhood undernutrition and growth faltering typically rely on targeting a standard set of risk factors that represent the immediate, underlying, and basic causes of stunting. The main multilevel conceptual framework used by the global nutrition community for the past 30 y is the UNICEF Undernutrition Conceptual Framework (8), upon which several variations have been based. One derivative developed by the WHO called “Childhood Stunting: Context, Causes and Consequences” summarizes 3 levels of factors associated with stunting, and is a product of the Healthy Growth Project (9). Adapted versions of the UNICEF framework were highlighted in the *Lancet* Series on Maternal and Child Nutrition in 2008 (10), and expanded in 2013 (1) to incorporate the theorized effects of both nutrition-sensitive and nutrition-specific interventions.

Although there are many theoretical determinants of stunting along the causal chain, only a subset has been studied well enough to quantify the strength of the relation. A recent comparative stunting risk-assessment analysis (11) grouped risk factors into 5 clusters: maternal nutrition and infection, teenage motherhood and short birth intervals, fetal growth restriction and preterm birth, child nutrition and infection, and environmental factors. With the exception of zinc supplementation trials in zinc-deficient children, all of the effect sizes for stunting risk were derived from meta-analyses of cohort studies or pooled analyses of Demographic and Health Survey (DHS) data. The leading global risk factors in terms of total number of attributable stunting cases were identified as follows: fetal growth restriction (defined as being born at term and small for gestational age), unimproved sanitation, childhood diarrhea, and maternal short stature.

Econometric analysis of underlying and basic determinants using data from 116 countries between 1970 and 2012 (12) identified several drivers of stunting reduction, including access to safe water, improved sanitation, gender equality, women's education, and nutritious food availability, with governance and income growth providing a supporting environment. Another cross-country analysis (13) of the developmental drivers of change in country-level nutritional status also highlighted asset ownership, health service access, maternal educational achievement, and lower fertility. However, growth in the economy and food production were key predictors only in countries experiencing food insecurity, and infrastructure was found to not be directly important to nutritional improvement.

A distinction must be made between analyses of the cross-sectional associations of specific determinants and stunting prevalence or mean HAZ and those that analyze the relative contribution of drivers of change in measures of child growth over time. Synthesizing the global evidence base detailing the latter type of analysis is prioritized in this article.

**Trends in indicators for key determinants in a set of top-performing countries**

Trends in key indicators for determinants of child stunting are depicted in Supplemental Figure 2 for the 13 top-performing countries described earlier. Overall, indicators have generally been improving over the last 30 y for the majority of top-performing countries, albeit unevenly. The rate of progress also varies widely by country. Despite these general improvements, at present there remain massive disparities in literacy rates, access to safe water and basic sanitation, and poverty rates between countries. Analyses of the relative contribution of these determinants to stunting reductions in a set of countries is described later in the article.

## Methods

**FIGURE 5:**
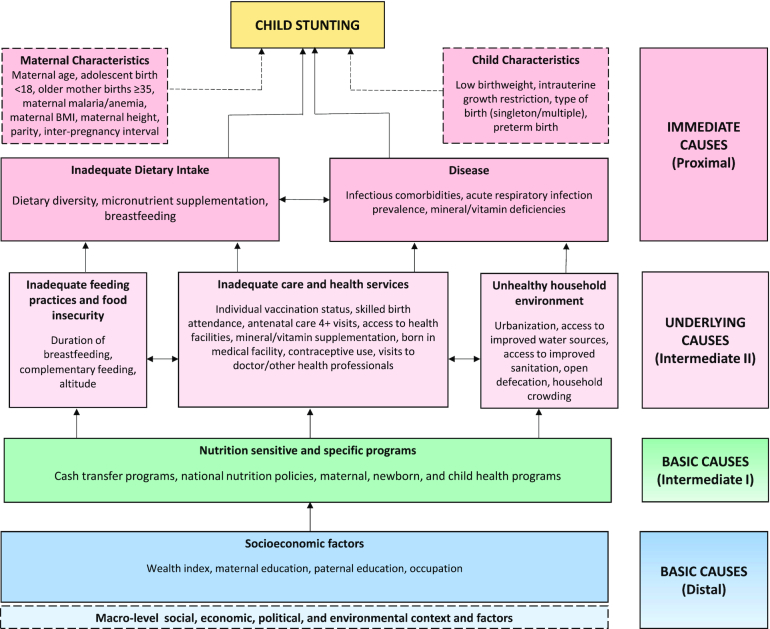
Conceptual framework of child stunting determinants. Determinants include those identified during the review process, and are based on those originally described in the UNICEF Undernutrition Conceptual Framework (8) and 2013 *Lancet* Maternal and Child Nutrition Series framework (1).

Building on existing frameworks and a mapping of key indicators and proxies from the global literature, we developed an adapted conceptual framework (**Figure 5**) to aid in the identification and interpretation of a variety of determinants of child stunting. Standard systematic review methods were used to identify and assess literature of interest. These included the development and execution of a search strategy in 15 databases, screening of titles and abstracts for relevance, followed by full-text screening against inclusion criteria and categorization of records. Additional studies were identified through gray literature searches, hand-searching of review reference lists, and update searches. Those records selected for inclusion were abstracted using a standardized form and underwent methodological quality appraisal. Abstracted data were then collated in tabular format, organized by determinant category and country, and narratively synthesized. The systematic review methods used are summarized in **Panel 3** and described in full in the **Supplemental Methods**.

Panel 3: **Review Methods**

Initial title and abstract screening of records was completed by a team of reviewers and focused on sensitivity and relevance. Studies were identified as potentially relevant if they met the 3 following inclusion criteria: *1*) a set of participants that included children <5 y was analyzed, *2*) ≥1 anthropometric outcome was measured, and *3*) the association between ≥1 stunting determinants and child growth outcomes was examined.

Subsequently, the full text of records was retrieved and reviewed, inclusion criteria were applied, and tags were assigned to the studies using a predefined algorithm, which was used to categorize included articles based on their study design. For the purposes of the current review, only the subset of studies examining the drivers of stunting decline or improvements in child growth outcomes at the national level were considered for full data abstraction. These studies contained analyses of multiple national cross-sectional surveys (e.g., DHS). For this subset of included studies, the prior categorization exercise was reassessed by a second reviewer to confirm eligibility for data abstraction. At this stage, the reference lists of reviews identified during the eligibility screening process were hand-searched for additional relevant studies for inclusion.

From the set of included national-level studies, quantitative and qualitative data were extracted, and methodological quality was appraised by the review team in duplicate. A standardized abstraction form was generated, which was designed to collect data on study characteristics, target population, outcome data, intervention/policy/program characteristics, and analysis methods. The estimates extracted included percentage contributions from decomposition analyses, regression coefficients, ORs, and RRs. In order to assess the quality of included studies based on their study design, we produced a tailored quality appraisal tool. We used a star rating system to assess quality across 4 domains: study design, sample selection, data sources, and statistical analyses measures. Abstracted data and quality appraisal ratings were matched between ≥2 reviewers, and any disagreements were resolved through discussion reaching a consensus.

Following the completion of data extraction, study variables were categorized into groups and subgroups based on the conceptual framework. The determinants/covariates were then mapped according to their conceptual domain grouping and subgrouping, and further study information was collated to assist with narrative synthesis.

### Ethics statement

As this was a systematic review of publicly available literature, ethical review was not required.

## Results

### Study selection

After database searches were executed (19 June 2018) and records exported and de-duplicated, a total of 16059 titles and abstracts were screened within Covidence, from which 2141 records were identified as potentially relevant. Full texts were retrieved and then screened against broad inclusion and exclusion criteria, which yielded a total of 1156 studies. Concurrently, all 1156 studies were then assigned a set of “tags” based on study design for further categorization. For the purposes of this work, the subset of studies that were assigned all of the following tags were included and abstracted: *1*) national-level or multinational-level, *2*) quantitative analysis, and *3*) analysis of trends over time (including ≥2 time points).

A total of 55 studies identified from the original indexed literature were assigned this set of tags and were eligible for inclusion in this systematic review. The reference lists of those studies tagged as “reviews” within Covidence were also screened, yielding an additional 4 studies not previously identified. Further gray literature searching done in February 2019 yielded 12 additional studies eligible for inclusion, and rapid catch-up searches for indexed literature done in May and August 2019 yielded an additional 6 and 12 studies, respectively. Thus, data included in this review were abstracted from a grand total of 89 discrete studies (see **Supplemental Figure 3** for a review flow diagram).

### Study characteristics and quality appraisal

The complete list of included studies (14–102), their characteristics, and quality appraisal scores can be found in **Supplemental Table 1**. The quality appraisal of included studies did not identify any meaningful differences in their individual methodological quality, nor raise any significant concerns that would affect the interpretation or synthesis of this set of observational studies. Additionally, groups and subgroups of determinants analyzed within the included studies are summarized in **Supplemental Table 2**.

### Synthesis of results from analyses of the drivers of improved child linear growth

The included studies analyzed data on the determinants of child growth and drivers of stunting reduction from >70 countries worldwide. A total 11 of studies (29, 30, 33, 40, 46, 48–51, 55, 99) contained data from national-level regression-decomposition analyses of change in HAZ and stunting prevalence in 14 countries (see **Table 1**). These studies best address our research question, and the following sections focus on synthesizing key findings from robust models across this set of studies, organized by groupings based on the basic, underlying, and immediate determinants of stunting. Other included studies using different methodologies are also described to augment the results.

**Table 1:** Summary of changes in stunting prevalence and HAZ statistically explained by changes in stunting determinant indicators within regression-decomposition analyses.

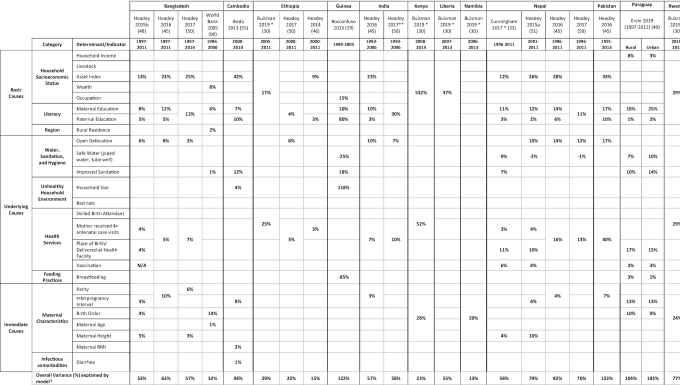

**Buisman 2019:** Maternal Risk (birth order, birth interval > 24 months, mothers taller than 150 cm, mother's age at birth)

**Ervin 2019:** ln(income), delayed vaccines, child breastfed at birth, ln(birth interval)

**Ikeda 2013**: Outcome is stunting prevalence, all other studies included outcome is HAZ

*0–23 months

**0–47 months

***0–10 years

^†^The total variance is the variance calculated by the study authors. Some models have adjusted for other covariates that have not been included in this table.

In a majority of these regression-decomposition studies (33, 40, 46, 48–51) multivariable regression and Oaxaca-Blinder decomposition methods were used to examine how different determinants predicted change in nutrition status. Multivariable linear regression and linear probability modeling were used to examine associations between HAZ and covariates of interest based on data collected regularly through DHS, the Multiple Indicator Cluster Survey (MICS), and other nationally representative surveys. The Oaxaca-Blinder decomposition is complementary to this initial regression analysis using the same individual/household-level data and ecological variables to assess predictors of HAZ or stunting change within a country between 2 survey time points at national or subnational levels. Some studies used extensions of Oaxaca-Blinder methods to incorporate dummy and nominal variables into the decomposition analysis (103, 104) or account for logit and probit models (105). Econometric analysis (89), quantile regression-decomposition (102), and calculation of the relative contribution to decreases in stunting prevalence (55) were other methods used by authors of included studies.

**Supplemental Table 3** summarizes the effect estimates from those studies that analyzed the associations between a variety of key indicators and the risk of stunting across multiple years. Estimates from decomposition analyses of changes in the population-level inequality of stunting at the national level are summarized in **Supplemental Table 4** and described in the **Supplemental Text**.

### Basic Stunting Determinants

#### Asset index

Household income is an important measure of a household's capacity to afford important elements related to improved nutrition such as food, water, sanitation, and medical care (12). Compared with other determinants, improvements in asset index consistently predicted some of the greatest improvements in HAZ across the countries analyzed. Of the total HAZ change observed in Cambodia (55) and Pakistan (49), 42% and 33% were attributed to asset index scores, respectively—the largest values analyzed. Similarly, improvements in asset index drove an estimated 25% of total HAZ change in Bangladesh (50).

#### Parental education

Maternal education is associated with decreased odds of stunting due to improvements in child health and care, and enhanced uptake and benefits from health interventions (1). Higher levels of paternal education are also associated with reduced odds of child stunting (106). Improvements in maternal educational attainment predicted 17% of the total HAZ change in Pakistan (49), between 11% and 14% in Nepal (33, 49–51), 10% in Guinea (29) and India (49), and 7% in Cambodia (55). Improvements in paternal education generally appeared to explain less HAZ change than maternal education, with the exceptions of Cambodia and Guinea. Increases in combined measures of parental education were estimated to predict 30% of the HAZ change in India (50).

### Underlying Stunting Determinants

#### Open defecation and sanitation

Environmental enteropathy and repeated diarrhea due to environmental fecal contamination and ingestion by young children—often related to widespread open-defecation practices or improper feces disposal—are theorized to increase the risk of stunting through reduced nutrient absorption and inflammation (107–110). Reductions in open defecation accounted for 17% of the total HAZ change in Pakistan (49), 10–14% in Nepal (49–51), 8% in Ethiopia (50), and 7–10% in India (49, 50). Similarly, improved sanitation infrastructure was found to be an important predictor of HAZ change in Cambodia (12%) (55), Guinea (18%) (29), and Nepal (7%) (33).

#### Access to improved water sources

The presence of a piped water source in the yard of a house is associated with water-related safe hygiene practices in mothers (111) and represents a pathway associated with diarrhea reduction (112). Improved access to safe water source predicted 7% of the change in HAZ in rural Paraguay (40) and 6% in Senegal (50).

A total of 40 included studies explored the association between childhood stunting and improved water sources (15, 21, 28–30, 33, 37, 39–41, 43, 46–51, 54, 55, 58, 59, 62, 66, 68–70, 74, 76, 77, 79, 83, 87, 89, 90, 93, 96, 98, 99, 101, 102). These studies used a variety of methods and examined associations with several indicators measuring access to clean water, including presence of improved sources, unimproved sources, and physical distance to water sources. There was variability in the significance and magnitude of the relation between improved sources of water and stunting.

#### Optimal antenatal care coverage and place of birth

High antenatal care coverage within a population is necessary to optimize maternal health and nutrition, as well as fetal growth and development. Evidence from a study of available health services in several LMICs demonstrated that a mother attending ≥4 antenatal care visits (ANC4+) with ≥1 visit with a skilled medical professional has been associated with a reduced risk of stunting (113). In addition, improved access to health care and skilled birth attendance at a health facility is associated with increased HAZ scores in children (114).

The extent to which improved antenatal care coverage predicted changes in child growth varied widely across the countries of interest. A combined measure of increases in coverage in ANC4+ and health facility births or skilled birth attendance accounted for 40%, 34%, and 29% of the change in HAZ in Pakistan (49), Senegal (50), and Rwanda (30), respectively. Associations between child growth and ANC4+, facility birth, or skilled attendance were analyzed in 14 different studies (19, 22, 23, 30, 33, 46, 48–51, 74, 75, 83, 93).

#### Bed nets

The largest predictor of stunting reduction and HAZ change in Zambia (35%) was the change in the proportion of households with bed nets (114), likely due in part to reductions in maternal malaria risk in the population and improved birth outcomes (115).

#### Vaccination coverage

High childhood vaccination coverage is an indicator of a functional health system. Improved vaccination coverage predicted between 4% (51) and 6% (33) of HAZ change in Nepal and 3% in Paraguay (40). A total of 11 studies (14, 22, 33, 40, 48, 51, 59, 68, 93, 99, 102) analyzed the relation between vaccination coverage and stunting.

#### Breastfeeding practices

In addition to being an optimal nutrition source, exclusive breastfeeding for the first 6 mo of life followed by continued breastfeeding for 2 y has a protective effect against diarrhea-related morbidity and mortality by reducing exposure to water-borne pathogens (116). Being breastfed at birth predicted 3% of the change in HAZ in rural Paraguay (40). There were 14 studies (21, 23, 29, 40, 43, 47, 55, 74, 81, 82, 87, 93, 98, 102) that explored the relation between improved breastfeeding practices and childhood stunting prevalence. Most of these analyses revealed significant associations between ever breastfeeding, breastfeeding duration, and child growth, although a handful of countries displayed nonsignificant relations, including Brazil, Dominican Republic, Honduras, Peru, and Sri Lanka.

#### Complementary feeding practices and food security

A total of 3 studies analyzed the associations between dietary intake and child growth outcomes including complementary food selection (82), actual micronutrient intake (25), and consumption of nonhuman milk (97). There were 4 studies that assessed the association between indicators of food insecurity and child growth (20, 62, 82, 100). However, none of these analyses were dynamic since they did not assess the relative predicted HAZ change over an interval.

### Immediate Stunting Determinants

#### Fertility

Family planning improves birth spacing and is important in preventing high-risk pregnancies among younger and older mothers, as well as women who have experienced closely spaced births (117). A longer time interval between births has been associated with lowered odds of stunting and reduced susceptibility to unfavorable outcomes for infants and children (118). Family-planning interventions may also reduce the number of children ever born to a mother, also known as parity (117). The association between fertility and stunting can be linked to the former's effect on preceding birth intervals (119), as longer birth intervals are thought to increase the amount of “nutrition-specific resources” available to individual children (118).

Declines in parity accounted for <7% of the observed HAZ change in the countries assessed (49, 50). While interpregnancy interval predicted 13% of HAZ change in Paraguay (40) and 8% of HAZ change in Cambodia (55), this value was only 3–4% across other countries analyzed (48–51).

A district-level multilevel ecological analysis in Peru (120) did not find a significant association between total fertility rate and stunting, whereas a pooled multicountry study (121) encompassing 23 countries found a significant association between fertility rate (births per 1000 women) and stunting. Three studies (22, 59, 70) examined the relation between childhood stunting and access to family planning including modern contraceptive use.

#### Maternal height

In a cross-country analysis of several LMICs, maternal height was found to be negatively correlated with stunting in infants and children, highlighting the importance of maternal nutrition and early-life factors on maternal growth and the effect on offspring (122).

There was considerable variability among the prediction estimates across countries analyzed. The largest estimates of maternal height predicting HAZ change were seen in Nepal, with values of 4% (33) and 10% (51) of HAZ change explained provided in separate analyses, while in Bangladesh these values were 3–5% (48, 50). In Rwanda, 24% was explained by a combined measure of maternal age, height, and interpregnancy interval (30).

#### Low birth weight

Being born with a low birth weight (<2500 g) can be an indicator of fetal growth restriction in utero, a process that can contribute to linear growth faltering. National analyses from Bangladesh (99), Malawi (37), Sri Lanka (82), and Uganda (101) examined the relation between a child's low birth weight and stunting as an outcome. In all studies, improved birth outcomes (i.e., increased birth weight or reduced low birth weight) were significantly associated with improved measures of child growth. However, only the Sri Lanka study used actual birth weight measured in the hospital, while others used a categorical subjective measure of relative size at birth.

#### Dietary diversity

Dietary diversity scores are used as an indicator of diet quality and density of micronutrients and macronutrients required for optimal growth and development. Inadequate dietary diversity is associated with increased odds of childhood stunting (123). One national study from Sri Lanka using multivariate regression did not find a significant relation between dietary diversity and child stunting (82), while a multinational study using logistic regression found that this relation was only significant in India (64).

#### Diarrhea

Diarrhea incidence has been found to be associated with stunting in young children, although findings have been inconsistent (124) and effect sizes are generally small (107). Diarrhea itself may not represent a direct cause of growth faltering, but rather, indicate enteric inflammation and dysfunction. Recent findings from the Etiology, Risk Factors and Interactions of Enteric Infections and Malnutrition and the Consequences for Child Health and Development (MAL-ED) birth cohort study (125) revealed that children with enteric pathogens had enteric inflammation and reduced linear growth, even when diarrhea was not present. Another recent cohort study (126) from Bangladesh found that diarrhea caused by certain pathogens was associated with linear growth but not all-cause diarrhea.

Reductions in diarrhea frequency predicted only 1% of HAZ change in Cambodia (55). Diarrhea was significantly associated with odds of stunting in Cambodia (55), Bangladesh (41), Malawi (37, 75), and Uganda (101), although the effect size varied. The relation between the incidence of diarrhea and growth outcomes in children was examined in 11 studies in total (14, 21, 35, 37, 41, 55, 62, 66, 75, 97, 98).

## Discussion

### Summary of evidence

Due to the very high heterogeneity and observational nature of the data within the included studies, the aim of this review is to identify broad patterns from existing national-level analyses examining how determinants of stunting can predict child growth outcomes. While the adapted tool used to assess the methodological quality of included studies did not identify meaningful differences in study quality, it is important to consider the percentage of statistically explained HAZ change described in studies with regression-decomposition analyses in the context of the total variance explained (see bottom row of Table 1), which can serve as an indicator of the strength of the model. For example, the models produced through regression-decomposition analyses for Ethiopia, Kenya, and Namibia have relatively low total variance explained. Rather than attempt to interpret individual estimates, this discussion highlights relatively large predicted values that arise consistently across multiple countries.

Among the basic determinants of stunting assessed, improvements in asset index score within households appeared to have the strongest explanatory power within national-level regression-decomposition analyses of the drivers of stunting reduction. This was especially true for several South Asian countries, Senegal, and Cambodia. Increasing parental educational levels was also found to be a consistently strong predictor of improvements in child growth outcomes.

Of the underlying determinants of stunting, reduction in the prevalence of open defecation and improved sanitation infra-structure were relatively important drivers of HAZ improvement in Cambodia, Guinea, India, Nepal, and Pakistan. Independent and combined measures of access to key maternal health services, including optimal antenatal care coverage and delivery in a health facility or with a skilled birth attendant, also accounted for substantially improved child growth, although the magnitude of variation explained differed substantially between countries.

Due to the unavailability of robust data collection for nutrition-specific factors within DHS and MICS datasets, there was less variety in the indicators representing the most immediate determinants of stunting, including dietary intake and birth outcomes. Several maternal characteristics predicted modest stunting reduction across the countries analyzed, including parity, interpregnancy interval, and maternal height.

Given the nature of these analyses, it is important to consider possible nonlinearity in some of the associations between determinants and child growth outcomes. For example, there appears to be a nonlinear relation between the prevalence of open defecation and mean HAZ scores within populations (48). This means that a 20 percentage point decrease in open-defecation prevalence from 80% to 60% compared with 30% to 10% may have very different impacts on child growth. This can potentially explain why reductions in open-defecation rates in Bangladesh predicted relatively less improvement in HAZ compared with other countries considered and suggests diminishing returns.

These findings are generally aligned with those from existing econometric analyses (12, 13) of the key drivers of stunting decline over the past few decades, including improvements in household asset index, parental education, health service access (ANC4+), and sanitation infrastructure. However, clear gaps in the evidence include those determinants where data availability and subsequent analyses were scarce, the most glaring of which are the lack of analyses on how dietary intake and diversity predict changes in nutrition status.

### Limitations

Due to the observational nature of the survey data discussed in the included studies, making causal inferences from the prediction values produced by regression-decomposition techniques is not possible. Additionally, many analyses had a very high proportion of unexplained variance or generated models that explained >100% of variation in HAZ change in a given country. This suggests that there may be other potential drivers of stunting reduction that have yet to be theorized, measured, or analyzed—some of which could be particularly important to the unique stories of stunting reduction in individual countries. The risk of omitted variable bias is a potential issue for the analyses of observational data, and a high percentage prediction value may represent a strong association but does not suggest a reduced risk of confounding. There are also potential limitations related to the datasets available, as we were not able to assess the quality of the stunting determinant indicator variables, nor the anthropometric data quality. Nevertheless, the regression-decomposition approach is relatively agnostic in its assessment of multiple stunting determinants at the national or subnational level, lending comprehensiveness and rigor to these analyses of observational data.

Not all LMICs were represented among the analyses discussed, and therefore this is not a globally exhaustive synthesis of the drivers of national stunting reduction. While there was good South Asian region representation, there was a particular lack of regression-decomposition analyses from countries in the African region. Sparse data from fragile and conflict settings limited our assessment of the determinants of stunting in these contexts. Future national-level explorations of the determinants of stunting may reveal additional important drivers of reduction. Despite having conducted thorough database searches, there remains the possibility of incomplete retrieval of studies that would have been eligible for inclusion and may have affected the interpretation of the overall results.

### Rationale for examining exemplars in stunting reduction with in-depth country case studies using mixed methods

Despite apparent progress on stunting reduction worldwide, regional trends do not illustrate the large variations in the rate of stunting reduction at the national level. Some countries have made excellent progress, while others lag behind. In order to refine our understanding of the drivers of changes in childhood linear growth faltering and generate meaningful and granular recommendations that countries can act upon, it is necessary to unpack the contributing factors surrounding these national variations in decline. In particular, it is helpful to focus analyses on periods of rapid national reductions in stunting prevalence in order to effectively determine the factors that accounted for these steep declines. This necessarily involves assessing which programs and policies have successfully predicted changes in coverage of key indicators.

Quantitative analyses of national survey data can provide an indication of which sectors were important to the national stunting-reduction story. However, in-depth country case studies using both quantitative and qualitative methods—folding in higher-resolution data on key indicators at the subnational level—can provide a more comprehensive and nuanced picture of the drivers of stunting reduction. This is especially important for examining within-country inequities in stunting reduction, which can be just as wide as the variation between countries in a given region. The other articles in this supplement issue describe the methods and results of in-depth case studies in 5 countries that have made exemplary progress in stunting reduction despite only modest economic growth.

### Conclusions

There are unique sets of stunting determinants that have predicted stunting reduction among countries that have reduced stunting, although there are several common drivers at the basic, underlying, and immediate level. Determinants identified to be particularly impactful include improvements in maternal and paternal education, household socioeconomic status, sanitation conditions, maternal health services access, and family planning. There is a need to conduct in-depth, retrospective, and mixed-methods case studies of determinants of stunting decline over multiple decades in order to overcome the limitations inherent in the existing literature and analyses of national survey data.

## Supplementary Material

nqaa159_Supplemental_Files

## Notes

This study was funded by a grant to the Centre for Global Child Health from Gates Ventures. The funder had no role in the design, implementation, analysis, or interpretation of the data.

Published in a supplement to The American Journal of Clinical Nutrition. The Guest Editor for this supplement was Mark Manary, and has no disclosures. The Supplement Coordinator for the supplement publication was Nadia Akseer, Gates Ventures/Hospital for Sick Children, Toronto, Canada. Supplement Coordinator disclosure: no conflicts to disclose. The Stunting Exemplars research Principal Investigator was Zulfiqar A Bhutta, Hospital for Sick Children, Toronto, Canada. Principal Investigator disclosure: no conflicts to disclose. Publication costs for this supplement were defrayed in part by the payment of page charges by Gates Ventures. The opinions expressed in this publication are those of the authors and are not attributable to the sponsors or the publisher, Editor, or Editorial Board of The American Journal of Clinical Nutrition.

Supplemental Methods, Supplemental Tables 1–4, Supplemental Figures 1–3, and Supplemental Text are available from the “Supplementary data” link in the online posting of the article and from the same link in the online table of contents at https://academic.oup.com/ajcn/.

SA, AS, and MS contributed equally.

Data described in the manuscript, code book, and analytic code will be made available upon request.

Abbreviations used: ANC4+, mother attended ≥4 antenatal care visits; DHS, Demographic and Health Survey; HAZ, height-for-age *z* score; LMIC, low- and middle-income country; MICS, Multiple Indicator Cluster Survey.
