# Supplementary material for: Stunting in childhood: an overview of global burden, trends, determinants, and drivers of decline
Source: Am J Clin Nutr. 2020 Aug 29;112(Suppl 2):777S–791S. doi: 10.1093/ajcn/nqaa159 (PMC7487433; doi:10.1093/ajcn/nqaa159)
Supplement: nqaa159_Supplemental_Files [file nqaa159_supplemental_files.zip › Online Supplementary Material FULL May112020.pdf]

Online Supplementary Material for: "Child stunting: an overview of global burden, trends, determinants, and drivers of decline"

Tyler Vaivada, Nadia Akseer, Selai Akseer, Ahalya Somaskandan, Marianne Stefopulos, Zulfiqar A Bhutta

Corresponding Author:
Professor Zulfiqar A. Bhutta, FRCPCH, PhD
Robert Harding Chair in Global Child Health & Policy
Centre for Global Child Health
The Hospital for Sick Children
Toronto, ON M5G 0A4, Canada
zulfiqar.bhutta@sickkids.ca

Table of Contents:

Supplementary Methods A – Detailed Systematic Review Methods --- **Page 2**
Supplementary Methods B – Search Strategy and Databases --- **Page 4**
Supplementary Methods C – Title/Abstract Screening Process and Criteria --- **Page 5**
Supplementary Methods D – Full Text Screening and Covidence Tagging Process --- **Page 6**
Supplementary Text - Summary of studies examining the determinants of stunting inequality --- **Page 8**
Supplementary References **--- Page 9**
Supplementary Table 1. Included Study Characteristics and References **--- Page 10**
Supplementary Table 2. Summary of stunting determinants and covariates assessed in analyses using national-level survey data **--- Page 26**
Supplementary Table 3 --- **See supplementary Excel file**
Supplementary Table 4. Summary of changes in stunting inequality predicted by changes in stunting determinant indicators **--- Page 29**
Supplementary Figure 1. National Stunting Prevalence **--- Page 30**
Supplementary Figure 2. Key indicator trends in top-performing countries **--- Page 32**
Supplementary Figure 3. Review flow diagram. **--- Page 33**

Supplementary Methods A

Eligibility criteria and study selection

Detailed information regarding the search strategy and databases consulted can be found in the Supplementary Methods B. Records identified through the peer-reviewed literature search were imported into and screened for eligibility, using the Covidence web-based software platform (Covidence systematic review software, Veritas Health Innovation, Melbourne, Australia. Available at www.covidence.org). Additionally, the following sources were hand-searched to identify relevant gray literature: African Development Bank, Alive & Thrive, Asian Development Bank, FAO, GAIN, IFPRI, Nutrition International, PAHO, UNDP, UNICEF, UNICEF INNOCENTI, WFP, WHO, World Bank Group Open Repository. Initial title and abstract screening of records was completed by a team of reviewers and focused on sensitivity and relevance (Supplementary Methods C). Studies were identified as potentially relevant if they met the three following inclusion criteria: i) a set of participants that include children under-5 was analyzed; ii) one or more anthropometric outcomes was measured; iii) the association between one or more stunting determinants and child growth outcomes was examined.

Subsequently, the full text of records was retrieved and reviewed, inclusion criteria were applied, and tags were assigned to the studies using a predefined algorithm (Supplementary Methods D), which was used to categorize included articles based on their study design. For a breakdown of the counts of studies in each category, including national-level cross-sectional studies and subnational-level studies using experimental, longitudinal cohort, and multiple cross-sectional designs, see Supplementary Figure 3. For the purposes of the current review, only the subset of studies examining the drivers of stunting decline or improvements in child growth outcomes at the national level were considered for full data abstraction. These studies contained analyses of multiple national cross-sectional surveys (e.g. Demographic and Health Survey). For this subset of included studies, the prior categorization exercise was reassessed by a second reviewer to confirm eligibility for data abstraction. At this stage, the reference lists of reviews identified during the eligibility screening process, were hand-searched for additional relevant studies for inclusion.

Data collection process

From the set of included national-level studies, quantitative and qualitative data were extracted, and methodological quality was appraised by the review team in duplicate. A standardized abstraction form was generated, which was designed to collect data on study characteristics, target population, outcome data, intervention/policy/program characteristics, and analysis methods. The measures of effect extracted included percent contributions from decomposition analyses, regression coefficients, odds ratios, and relative risks. Select decomposition analyzes were recalculated by the reviewers to account for the explained/unexplained percentage of a given model.

Quality appraisal

In order to assess the quality of included studies based on their study design, we produced a tailored quality appraisal tool by adapting two pre-existing tools: the Newcastle-Ottawa Scale for Cohort Studies (1) and the NIH Quality Assessment Tool for Observational Cohort and Cross-Sectional Studies (2). We used a star rating system to assess quality across four domains: study design, sample selection, data sources, and statistical analyses measures. Abstracted data and quality appraisal ratings were matched between at least two reviewers, and any disagreements were resolved through discussion reaching a consensus. The star ratings were then converted to their corresponding number score (i.e. 2 stars = 2 points, 3 stars = 3 points, 4 stars = 4 points). The final score for each study was derived by calculating an average of the scores across all categories. The full list of quality appraisal scores can be found in Supplementary Table 1.

Synthesis of results

Following the completion of data extraction, study variables were categorized into groups and subgroups based on the conceptual framework. The determinants/covariates were then mapped according to their conceptual domain grouping and subgrouping, and further study information (author, year, study dates, total population (N), country, outcome, effect sizes) was collated to assist with narrative synthesis.

Supplementary Methods B

Generic Search Strategy

1. "stunt*" OR "linear growth" OR "growth faltering" OR "HAZ" OR "height-for-age" OR "LAZ" OR "length-for-age" OR "undernutrition" or "malnutrition" OR "stature"
2. "child*" or "infan*" or "under-five" or "under-5" or "0-59 months" or "0-24 months" or "0-36 months" or "6-59 months" or "24-59 months" or "6-24 months"
3. "determinant*" OR "polic*" OR "program*" OR "intervention*" OR "factor*" OR "predictor*" OR "initiativ*" OR "strateg*" OR "correlat*"
4. "trend*" OR "longitudinal" OR "reduc*" OR "trajector*" OR "chang*" OR "declin*"
5. 1 AND 2 AND 3 AND 4

Databases Searched

- MEDLINE
- Embase
- AMED
- CAB Abstracts
- CINAHL
- Cochrane CENTRAL
- Campbell Collaboration
- EPPI Centre Trials Register (TRoPHI)
- 3ie
- JOLIS
- African Journals Online
- WHOLIS
- LILACS
- Scopus
- Web of Science

Grey Literature Sources

Searching was done using Google, hand searching of reference lists of relevant reviews and directly searching relevant organization websites, including:

- National, regional and headquarter websites for UNICEF, WHO, UNDP, WFP, FAO
- World Bank Group Open Knowledge Repository
- Asian Development Bank
- African Development Bank
- Nutrition International
- Global Alliance for Improved Nutrition
- International Food Policy Research Institute

Supplementary Methods C

Title/Abstract Screening Process and Criteria:

- *Focus on sensitivity - collecting all potentially relevant records*
- Screen title + abstract for relevance:
    - Studies includes under-5 population
    - Measured anthropometric outcomes (e.g. height, length, weight, HAZ, LAZ, WAZ, stunting, underweight, etc.)
    - Analyzes the association/correlation/contribution of one or more stunting determinants, including but not limited to:
        a. Nutrition specific/sensitive programs or policies
        b. Food security
        c. Dietary intake
        d. Environmental conditions
        e. Health service utilization
        f. Childhood vaccines
        g. Infectious disease (e.g. pneumonia, diarrhea)
        h. Improved WASH
        i. Improved maternal literacy
        j. Changes in fertility
        k. Decrease SGA/PTB
        l. Maternal/child characteristics or genetics
        m. Parity
        n. Intergenerational effects
        o. Gender
        p. Socioeconomic status

Supplementary Methods D

Full Text Screening and Covidence Tagging Process

<table>
<tr><th>Full Text Screening Questions and Tagging Process</th><th>Yes</th><th>No</th></tr>
<tr><td>1. Covers an under-5 population?</td><td>Move to 2. ↲</td><td rowspan="3">Exclude study with reason ×</td></tr>
<tr><td>2. Reports on one or more relevant anthropometric outcomes? (e.g. stunting, height, length, HAZ, LAZ)</td><td>Move to 3. ↲</td></tr>
<tr><td>3. Discusses the association/correlation/ contribution of one or more stunting determinants (see details below), e.g.
a. Nutrition-specific or nutrition-sensitive, policy, strategy, program, or intervention implemented to address chronic undernutrition in children, or one or more of its determinants.
b. Contextual factor affecting one or more stunting determinants</td><td>Move to 4. ↲</td></tr>
<tr><td><u>4. Assign appropriate tags (see details below):</u>

Tagging algorithm:
1. National or subnational?
2. Quantitative or qualitative/narrative?
3. Change over time (2+ time points) or single cross sectional?
4. If subnational, quantitative, analyzing change over time, choose one:
a. 2 or more cross sectional studies
b. RCTs/quasi-experimental
c. Longitudinal cohort</td><td colspan="2" rowspan="2"></td></tr>
<tr><td>5. Include study</td></tr>
</table>

Interventions and contextual factors can include, but are not limited to:

- **Nutrition specific/sensitive programs or policies**
  - E.g. those in Hossain et al.(3)(see right)
- **Food security**
- **Dietary intake**
- **Environmental conditions**
- **Health service utilization**
- **Childhood vaccines**
- **Infectious disease (e.g. pneumonia, diarrhea)**
- **Improved WASH**
- **Improved maternal literacy**
- **Changes in fertility**
- **Decrease SGA/PTB**
- **Maternal/child characteristics or genetics**
- **Parity**
- **Intergenerational effects**
- **Gender**
- **Socioeconomic status**

Nutrition-specific interventions and programs

a) Health and nutrition during adolescence, preconception, pregnancy and lactation
b) Maternal dietary or micronutrient supplementation
c) Promotion of optimum breastfeeding
d) Complementary feeding and responsive feeding practices and feeding stimulation
e) Dietary supplementation, food diversification and micronutrient supplementation or fortification for children
f) Treatment of severe acute malnutrition
g) Disease prevention and management
h) Nutrition in emergencies

Nutrition-sensitive interventions and programs

a) Agriculture and food security
b) Social safety nets
c) Early child development
d) Maternal mental health
e) Women's empowerment
f) Child protection
g) Schooling
h) Water, sanitation and hygiene
i) Health and family planning services

If study meets inclusion criteria, assign up to 4 tags in Covidence:

1. National-level OR Multi-national-level
   a. Quantitative analysis (association/correlation/contribution)
      i. Change over time (2+ time points)
      ii. Single cross-sectional (1 time point)
   b. Qualitative synthesis/narrative only
2. Subnational-level
   a. Quantitative analysis (association/correlation/contribution)
      i. Change over time (2+ time points)
         1. 2+ cross sectional (e.g. panel datasets)
         2. RCTs + quasi-experimental studies (e.g. pre-post)
         3. Longitudinal cohort
      ii. Single cross-sectional (1 time point)
   b. Qualitative synthesis/narrative only

Determinants of stunting inequality

There were 11 studies (4-14) identified that contained data from decomposition analyses of changes in the population-level inequality of stunting 14 countries (**Supplementary Table 4**). Rather than assessing how various stunting determinants can predict changes in mean HAZ in a population of children, these studies used multiple methods to assess how these determinants modified patterns of disparity in linear growth outcomes within a population of children (e.g. the difference in HAZ between those children who live in households with the lowest and highest wealth index score). As with the regression-decompositions of change in HAZ, wealth index score was a consistently large predictor of unequal stunting distributions across the countries assessed. Maternal education, nutritional status and parity also accounted for substantial child growth disparities, along with delivery at a health facility. The relative importance of access to safe water and sanitation to stunting inequalities varied between countries.

Supplementary References

1. Wells G, Shea B, O'Connell D, Peterson J, Welch V, Losos M, P T. Newcastle-Ottawa quality assessment scale cohort studies. 2019. [Available from: http://www.ohri.ca/programs/clinical_epidemiology/oxford.asp.

2. NIH National Heart Lung and Blood Institute. Study Quality Assessment Tools. 2019. [Available from: https://www.nhlbi.nih.gov/health-topics/study-quality-assessment-tools.

3. Hossain M, Choudhury N, Abdullah KAB, Mondal P, Jackson AA, Walson J, Ahmed T. Evidence-based approaches to childhood stunting in low and middle income countries: a systematic review. Archives of disease in childhood. 2017;102(10):903-9.

4. Huda TM, Hayes A, El Arifeen S, Dibley MJ. Social determinants of inequalities in child undernutrition in Bangladesh: A decomposition analysis. Matern Child Nutr. 2018;14(1).

5. Rabbani A, Khan A, Yusuf S, Adams A. Trends and determinants of inequities in childhood stunting in Bangladesh from 1996/7 to 2014. Int J Equity Health. 2016;15(1):186.

6. Amarante V, Figueroa N, Ullman H. Inequalities in the reduction of child stunting over time in Latin America: evidence from the DHS 2000-2010. Oxford Development Studies. 2018;46(4):519-35.

7. Nie P, Rammohan A, Gwozdz W, Sousa-Poza A. Changes in Child Nutrition in India: A Decomposition Approach. Int J Environ Res Public Health. 2019;16(10):22.

8. Angdembe MR, Dulal BP, Bhattarai K, Karn S. Trends and predictors of inequality in childhood stunting in Nepal from 1996 to 2016. Int J Equity Health. 2019;18(1):42.

9. Akombi BJ, Agho KE, Renzaho AM, Hall JJ, Merom DR. Trends in socioeconomic inequalities in child undernutrition: Evidence from Nigeria Demographic and Health Survey (2003 - 2013). PLoS One. 2019;14(2):e0211883.

10. Ervin PA, Bubak V. Closing the rural-urban gap in child malnutrition: Evidence from Paraguay, 1997-2012. Econ Hum Biol. 2019;32:1-10.

11. Kien VD, Lee HY, Nam YS, Oh J, Giang KB, Van Minh H. Trends in socioeconomic inequalities in child malnutrition in Vietnam: findings from the Multiple Indicator Cluster Surveys, 2000-2011. Glob Health Action. 2016;9:29263.

12. O'Donnell O, Nicolas AL, Van Doorslaer E. Growing richer and taller: Explaining change in the distribution of child nutritional status during Vietnam's economic boom. Journal of Development Economics. 2009;88(1):45-58.

13. Wagstaff A, van Doorslaer E, Watanabe N. On decomposing the causes of health sector inequalities with an application to malnutrition inequalities in Vietnam. Journal of Econometrics. 2003;112(1):207-23.

14. Hangoma P, Aakvik A, Robberstad B. Explaining changes in child health inequality in the run up to the 2015 Millennium Development Goals (MDGs): The case of Zambia. PLoS One. 2017;12(2):e0170995.

Supplementary Table 1. Included Study Characteristics

Geographic Region	Country	Primary Author, Year	Title	Source	Data Source	Study Population	Time Period	Determinant Categories	Covariate/indicator included in analysis	Analysis Method	Outcome	Baseline Child Growth Indicator	Endline Child Growth Indicator	Quality Appraisal Score
Sub-Saharan Africa	Nigeria	Adesugba, 2018	Child nutritional status, welfare and health in Nigerian households	Gray Literature	DHS 2003, 2008, 2013	Reproductive-age women (15-49) and their children (<60 months)	2003-2013	Household SES, Literacy, Maternal Characteristics, Unhealthy Household Environment, Child Characteristics, Infectious Diseases & Morbidities, Health Services	Household wealth index, educational level, respondents age, household size, gender of household head, occupation, presence of extra children, child's age, gender of child, maternal BMI, incidence of diarrhea and fever, access to health facility, vaccine status	Multilevel Mixed-Effects Probit Analysis	Stunting Prevalence	Stunting Incidence: 41%	Stunting Incidence: 41%	3.21
South Asia	Nepal	Adhikari, 2019	Determinants of stunting among children aged 0–59 months in Nepal: findings from Nepal Demographic and Health Survey, 2006, 2011, and 2016	Updated Search	DHS 2006, 2011, 2016	children aged 0–59 months	2006-2016	Unhealthy Household Environment, Parental Characteristics, Child Characteristics, Household SES, WASH, Maternal Characteristics, Literacy, Household SES, Birth Outcomes, Infectious Diseases & Morbidities	Family size, Headship of households, Caste/ethnicity, Wealth quintile, Place of residence, Household food security status, Access of drinking water, Access of toilet, Age of mother, Years of schooling of mother, Number of living children, Employment, Mother BMI, Mother anemia, Age of child, Sex of child, Birth Order, Size at time of birth, Anemia	Logistic Regression	Stunting	Stunting Prevalence: 49.3%<br><br>HAZ: -1.93	Stunting Prevalence: 35.8%<br><br>HAZ: -1.29	3.21
Multi-Region	22 countries in low- and middle-income	Adjaye-Gbewonyo, 2019	Agricultural trade policies and child nutrition in low- and middle-income countries: a cross-national analysis	Updated Search	DHS 1991-2010, World Bank's Updated National and Global Estimates of Distortions to Agricultural Incentives, 1955 to 2011 World Development Indicators dataset, KOF Index of Globalization and Polity IV dataset	children aged 6 to 35 months	1991-2010	Household SES,	Non-tradeable agriculture, Parental occupation, Share of tradeable agriculture	Pooled Fixed-Effects Regression	HAZ			3.21
Sub-Saharan Africa	Nigeria	Akombi, 2019	Trends in socioeconomic inequalities in child undernutrition: evidence from Nigeria Demographic and Health Survey (2003-2013)	Updated Search	DHS 2003, 2008, 2013	Children aged 0-59 months	2003-2013	Child Characteristics, Maternal Characteristics, Literacy, Household SES, Region	Child's age, mother's age, sex of child, maternal working status, maternal education, father's education, household wealth index, type of residence, geopolitical zone	Logistic Regression<br><br>Decomposition - Concentration Index	Stunting<br><br>Stunting Inequality	Stunting Prevalence: 45.0%	Stunting Prevalence: 36.8%	3.29
Sub-Saharan Africa	Burkina Faso, Ghana, Mali, Nigeria, Senegal	Alfani, 2015	Vulnerability to Malnutrition in the West African Sahel	Gray Literature	Burkina Faso: DHS 2003, 2010<br>Ghana: DHS 2003, 2008<br>Mali: DHS 2001, 2006<br>Nigeria: DHS 2003, 2008<br>Senegal: DHS 2005, 2010	Children who were born 1-3 years before each survey	2000-2012	Macro level: Environmental and Community Context, Region, Unhealthy Household Environment, Literacy, Household SES, Parental Characteristics	Shock, rainfall levels, type of residence, number of household members, educational level, household assets, condition of dwelling floor, age of household head, gender of household head, child sex, child is twin	Difference-in-Difference Pooled Regression	Stunting Vulnerability			3.21
Middle East & North Africa South Asia	Afghanistan<br>Bahrain<br>Djibouti<br>Egypt<br>Iran<br>Iraq<br>Jordan<br>Kuwait<br>Lebanon<br>Libya<br>Morocco<br>Oman<br>Pakistan<br>Qatar<br>Saudi Arabia<br>Somalia<br>Sudan<br>Syria<br>Tunisia<br>United Arab Emirates<br>Yemen	Almasi, 2019	Investigation of some Factors Affecting Stunting and Wasting among the Under-Five Children in Eastern Mediterranean Region	Updated Search	WHO Global Health Observatory (GHO)<br>World Meteorological Organization (WMO)	Children under the age of five	2005-2016	Household SES, Maternal Characteristics, Health Services	Reproductive, maternal, newborn and child health interventions, Economic status, Life expectancy at birth, Average wind speed, Prevalence of anemia in pregnant women, Births attended by skilled health personnel	Graphical Statistical Analysis<br><br>Stepwise Regression<br><br>Path Analysis and Cluster Analysis	Stunting Prevalence			2.86
Sub-Saharan Africa	Uganda	Amaral, 2017	Using the Uganda National Panel Survey to analyze the effect of staple food consumption on undernourishment in Ugandan children	Covidence	Uganda National Panel Survey 2009/10, 2010/11, 2011/12	Children	2009-2012	Food Security, Household SES, Child Characteristics, Unhealthy Household Environment, Parental Characteristics, Literacy, Region	Staple food spending, food insecurity, total household spending, gender of household head, age, household occupants, percent of adult females, type of residence, father and mother present, education of household head, child sex	Binary Logistic Regression	Stunting			3.36

Latin America & Caribbean	Bolivia<br>Brazil<br>Colombia<br>Dominican Republic<br>Haiti<br>Honduras<br>Peru	Amarante, 2018	Inequalities in the reduction of child stunting over time in Latin America: evidence from the DHS 2000–2010	Covidence	Bolivia: DHS 2003, 2008<br>Brazil: DHS 1996, 2006<br>Colombia: DHS 2005, 2010<br>DR: DHS 2002, 2007<br>Haiti: DHS 2006, 2012<br>Honduras: DHS 2005, 2011<br>Peru: DHS 2007, 2012	Women 15–49 years of age and on their children under five years of age	Bolivia: 2003-2008<br>Brazil: 1996-2006<br>Colombia: 2005-2010<br>DR: 2002-2007<br>Haiti: 2006-2012<br>Honduras: 2005-2011<br>Peru: 2007-2012	Child Characteristics, Region, Maternal Characteristics, Literacy, Household SES, Dietary Intake, WASH, Infectious Diseases & Morbidities	Child age, sex, type of residence, ethnicity, mother's weight, mother's education, mother's age, number of children, wealth index, breastfeeding practices, water & sanitation, incidence of diarrhea	Regression<br><br>Concentration Curve<br><br>Concentration Index<br><br>Decomposition - Concentration Index	HAZ<br><br>Stunting Inequality			3.14
Sub-Saharan Africa	Ethiopia	Ambel, 2017	Examining changes in maternal and child health inequalities in Ethiopia	Covidence	DHS 2000, 2005, 2011, 2014	Women 15–49 years of age and on their children 0-59 months	2000-2004	Health Services	Measles vaccination, full immunization, contraceptive prevalence, antenatal care, skilled birth attendant	Difference-in-Difference Analysis<br><br>Decomposition - Concentration Index	Stunting Inequality	Stunting Prevalence: 57.0%	Stunting Prevalence: 40.6%	3.14
South Asia	Nepal	Angdembe, 2019	Trends and predictors of inequality in childhood stunting in Nepal from 1996 to 2016	Updated Search	DHS 1996, 2001, 2006, 2011, 2016	All children under-three years of age born in the three years before the survey to women interviewed (DHS 1996)<br><br>All children under-five in the household (DHS 2001, 2006, 2011, 2016	1996-2016	Household SES, Literacy, Maternal Characteristics, Health Services, Child Characteristics, Dietary Intake, Birth Outcomes	Wealth quintiles, Mother's education, Husband/Partner's education, Maternal short stature, Mother's BMI, Antenatal care visits, Delivery in health facility, Children aged 25-59 months, Birth order, Breastfeeding, Perceived size of baby at birth, Caste/ethnicity	Probit Regression<br><br>Decomposition - Concentration Index	Stunting Inequality	Stunting Prevalence: 56.6%	Stunting Prevalence: 35.8%	3.21
South Asia	Bangladesh	Bagmar, 2015	Tracking changes and identifying determinants of child malnutrition status over the past decade in Bangladesh	Covidence	DHS 2004, 2007, 2011	Children age 0-5 years	2004-2011	Child Characteristics, Maternal Characteristics, Region, Household SES, WASH	Sex of the child, child's age, birth order, mothers age, mother's education, working status, place of residence, division, religion, media exposure, poverty, toilet facility	Pearson's Chi-Square<br><br>Binary Logistic Regression	Stunting	Stunting Prevalence: 49.7%	Stunting Prevalence: 40.9%	3.14
North America	USA	Ballew, 1999	Blood lead concentration and children's anthropometric dimensions in the Third National Health and Nutrition Examination Survey (NHANES III), 1988-1994	Covidence	Third National Health and Nutrition Examination Survey, 1988-1994	Non-Hispanic white, non-Hispanic black, and Mexican-American children age 1 to 7 years	1988-1994	Child Characteristics, Dietary Intake, Household SES	Sex, ethnic group, child age, iron status, dietary intake, medical history, sociodemographic factors, and household characteristics, nutrition	Multiple Regression	Height (cm)			3.29
Sub-Saharan Africa	Côte d'Ivoire	Barankanira, 2017	Stunting among children under 3 years of age in Côte d'Ivoire: spatial and temporal variations between 1994 and 2011	Covidence	DHS 1994, 1998, 2011	Children 0-35 months	1994-2011	Region, Child Characteristics, Literacy, Maternal Characteristics, Household SES	Geographical zone, sex, age group, birth order, level of education, mother's BMI, wealth index	Multilevel Multivariable Logistic Regression	Stunting	Stunting Prevalence: 30.7%	Stunting Prevalence: 28.7%	3.21
Sub-Saharan Africa	Ethiopia	Biadgilign, 2016	Does economic growth reduce childhood undernutrition in Ethiopia?	Covidence	DHS 2000, 2005, 2010	Children aged 0-59 months	2000-2010	Child Characteristics, Maternal Characteristics, Parental Characteristics, Region, Household SES, WASH, Unhealthy Household Environment, Literacy	Child's age, sex, age of women, region, place of residence, sex of household head, wealth index, type of toilet facility, source of drinking water, maternal height, respondent's occupation, partner's occupation, number of household members, number of children under 5 in household, partner's education level	Multilevel Mixed Logistic Regression	Stunting			3.29
Sub-Saharan Africa	Ethiopia	Biadgilign, 2019	Good governance, public health expenditures, urbanization and child undernutrition Nexus in Ethiopia: an ecological analysis	Updated Search	DHS 2000, 2005, 2011, 2016	Children 6-59 months	2000-2016	Macro Level: Political Context, Region, Child Characteristics, Literacy, Household SES, WASH, Unhealthy Household Environment, Maternal Characteristics	Government effectiveness, regulatory quality, control corruption, region, sex, partner's education level, current age of child, wealth index, toilet type, drinking source, residence, highest educational level of the respondents, number of children under five, sex of household head, respondent's occupation, partner's occupation, parity, age of women, number of household members	Univariate and Multivariate Regression	Stunting	Stunting Prevalence: 53.30%	Stunting Prevalence: 34.20%	3.14

South Asia	Nepal	Bin Nisar, 2016	Iron-Folic Acid Supplementation During Pregnancy Reduces the Risk of Stunting in Children Less Than 2 Years of Age: A Retrospective Cohort Study from Nepal	Covidence	DHS 2001, 2006, 2011	Children age <2 years	2001-2011	Region, Maternal Characteristics, Literacy, Household SES, Unhealthy Household Environment, WASH, Birth Outcomes, Child Characteristics, Dietary Intake, Infectious Diseases & Morbidities, Health Services	Ecological region, place of residence, maternal marital status, maternal religion, maternal educational status, maternal occupation, paternal educational status, paternal occupation, fuel used for cooking, source of drinking water, sanitation facilities, pooled household wealth index, maternal age at childbirth, maternal desire for pregnancy, maternal smoking status, maternal height, maternal perception of birth size, birth status, birth rank and interval, sex of child, timing of initiation of breastfeeding, age of child, incidence of diarrhoea, antenatal care	Multivariate Poisson Regression	Stunting	Stunting Prevalence: 47.1%	Stunting Prevalence: 17.6%	3.29
Sub-Saharan Africa	Guinea	Boccanfuso, 2013	A new avenue for understanding the nutritional status of children in Guinea	Covidence	DHS 1999, 2005	Children 0-59 months	1999-2005	Child Characteristics, Birth Outcomes, Dietary intake, Infectious Diseases & Morbidities, Maternal Characteristics, Literacy, Household SES, Unhealthy Household Environment, Parental Characteristics, WASH, Region	Child's age, gender, twin status, birth order, breast-feeding, incidence of diarrhea, cough, fever, mother's age at childbirth, mother's height, mother's marital status, literacy, employment status, household size, number of children under five, gender of household head, partner education, sanitation, water, electricity, region, type of residence, ethnicity	Ordinary Least Squares (OLS)<br><br>Decomposition	HAZ	HAZ: -1.21	HAZ: -1.45	3.21
Sub-Saharan Africa	Ethiopia<br>Ghana<br>Kenya<br>Liberia<br>Namibia<br>Niger<br>Rwanda<br>Sierra Leone<br>Zambia	Buisman, 2019	What explains the fall in child stunting in Sub-Saharan Africa?	Updated Search	Ethiopia: DHS 2005, 2011<br>Ghana: DHS 2008, 2014<br>Kenya: DHS 2008, 2014<br>Liberia: DHS 2007, 2013<br>Namibia: DHS 2006, 2013<br>Niger: DHS 2006, 2012<br>Rwanda: DHS 2010, 2014<br>Sierra Leone: DHS 2008, 2013<br>Zambia: DHS 2007, 2013	Children 0-23 months	Ethiopia: 2005-2011<br>Ghana: 2008-2014<br>Kenya: 2008-2014<br>Liberia" 2007-2013<br>Namibia: 2006-2013<br>Niger: 2006-2012<br>Rwanda: 2010-2014<br>Sierra Leone: 2008-2013<br>Zambia: 2007-2013	Health Services, Literacy, Household SES, WASH, Child Characteristics, Region, Macro Level: Environmental and Community Context	Antenatal care, delivered by skilled birth attendant, maternal education, paternal education, wealth index, water, sanitation, birth order, birth interval, maternal height, mother's age, livestock, type of residence, child's age, sex, season	Linear Regression<br><br>Decomposition	HAZ<br><br>Stunting	HAZ:<br>Ethiopia: -1.25<br>Ghana: -0.51<br>Kenya: -1.11<br>Liberia: -0.90<br>Namibia: -0.98<br>Niger: -1.51<br>Rwanda: -1.34<br>Sierra Leone: -0.84<br>Zambia: -1.32<br><br>Stunting Rate:<br>Ethiopia: 0.38<br>Ghana: 0.20<br>Kenya: 0.32<br>Liberia: 0.27<br>Namibia: 0.27<br>Niger: 0.42<br>Rwanda: 0.35<br>Sierra Leone: 0.29<br>Zambia: 0.39	HAZ:<br>Ethiopia: -1.04<br>Ghana: -0.57<br>Kenya: -0.87<br>Liberia: -0.69<br>Namibia: -0.47<br>Niger: -1.16<br>Rwanda: -1.23<br>Sierra Leone: -0.94<br>Zambia: -1.27<br><br>Stunting Rate:<br>Ethiopia: 0.30<br>Ghana: 0.14<br>Kenya: 0.22<br>Liberia: 0.22<br>Namibia: 0.16<br>Niger: 0.32<br>Rwanda: 0.32<br>Sierra Leone: 0.32<br>Zambia: 0.37	3.29
Sub-Saharan Africa	Ethiopia	Christiaensen, 2001	Child malnutrition in Ethiopia: can maternal knowledge augment the role of income?	Covidence	Welfare Monitoring Surveys (WMS) 1995/1996, 1997, 1998<br>Household income and expenditure survey (HICES) 1995/1996<br>Health and Nutrition Survey<br>(HNS) 1998	Households with children 3-60 months	1996-1998	Child Characteristics, Birth Outcomes, Women's Empowerment, Literacy, Household SES, WASH, Health Services, Region	Sex, Child Age (Months), Child Is Twin, Household Size, Female Headed Household, Highest Grade Completed By Most Educated Female Adult, Info On Female Education, Highest Grade Completed By Most Educated Male Adult, Post Secondary Education Most Educated Adult, Info On Male Adult Education, Log Real Household Expenditure Per Adult Equivalent, Non-Self Proportion households/Cluster with Flush Toilets, Distance To Nearest Health Center, Distance To Nearest Health Center (.5km), Non-Self Proportion households/Cluster Who Own Radio, Non-Self Proportion households/Cluster Who Own TV, Rural, Enset Producing Zones	Linear Regression	HAZ	Stunting Prevalence: 66%	Stunting Prevalence: 55%	3.14
South Asia	India	Corsi, 2015	No Female Disadvantage in Anthropometric Status among Children in India: Analysis of the 1992-1993 and 2005-2006 Indian National Family Health Surveys	Covidence	Indian National Family Health Survey (NFHS) 1992/2003, 2005/2006	Children 0-47 months	1992-2006	Child Characteristics	Gender of child, birth order	Linear and Logistic Regression	HAZ			3.21
South Asia	Nepal	Cunningham, 2017	Maternal and Child Nutrition in Nepal: Examining drivers of progress from the mid-1990s to 2010s	Covidence	DHS 1996, 2001, 2006, 2011<br>Qualitative interviews	Children 0-2 years and 20 mothers living in rural,	1996-2011	Maternal Characteristics, Household SES, Literacy, Health Services, Dietary Intake, Child Characteristics, Macro	Maternal BMI, asset index, maternal education, paternal education, antenatal care, iron supplementation, born in health facility, all vaccinations, birth order, birth interval, community	Narrative Analysis of open-ended interviews	LAZ	LAZ: -1.89<br><br>Stunting Prevalence: 47.8%	LAZ: -1.16<br><br>Stunting Prevalence: 27.0%	3.14

						remote communities		Level: Environmental and Community Context, WASH, Women's Empowerment	toilet use, water source, maternal height, mother's age, mother's empowerment	Linear and Logistic Regression				
Sub-Saharan Africa<br>Middle East & North Africa	30 countries	Darrouzet-Nardi, 2017	Nonviolent civil insecurity is negatively associated with subsequent height-for-age in children aged <5 y born between 1998 and 2014 in rural areas of Africa	Covidence	DHS surveys 1998-2014	Child <5 with sibling, and mother between the age of 15-49	1998-2014	Child Characteristics, Maternal Characteristics, Infectious Diseases & Morbidities	Child age, gender, child is first born, short birth interval, child was sick	Pooled OLS	HAZ			3.29
South Asia	India	Das Gupta, 2005	Improving Child Nutrition Outcomes in India : Can the Integrated Child Development Services Program Be More Effective?	Gray Literature	NFHS 1992/93, 1998/99	Children aged 0-4 in the 1992 survey and 0-3 in the 1998 survey	1992-1998	Other Programs/Policies/Interventions	ICDS Program	Propensity Score Method	HAZ			3.07
Multi-Region	67 low- and middle-income countries (LMICs)	daSilva, 2018	Socioeconomic Inequalities Persist Despite Declining Stunting Prevalence in Low- and Middle-Income Countries	Covidence	Demographic Health Surveys (DHSs) and Multiple Indicators Clusters Surveys (MICSs)	Children <5 y of age (under-5)	1993-2015	Household SES	Wealth quintiles	Pooled Linear Multilevel Regression<br><br>Slope Index of Inequality<br><br>Concentration Index of Inequality	Stunting Prevalence	Stunting Prevalence: 50.0%	Stunting Prevalence: 34.4%	3.21
Sub-Saharan Africa	Malawi	Doctor, 2017	Trends and Determinants of Child Growth Indicators in Malawi and Implications for the Sustainable Development Goals	Covidence	DHS 1992, 2000, 2004, 2010, 2015-16	Children under-5 years of age	1992-2016	Region, Household SES, WASH, Maternal Characteristics, Literacy, Unhealthy Household Environment, Child Characteristics, Birth Outcomes, Infectious Diseases & Morbidities	Residence, region of residence, wealth index, source of drinking water, toilet facilities, mother's age, highest educational level, number of under-5 children living with mother, child's sex, age, birth order, size at birth, incidence of fever, diarrhoea, cough	Logistic Regression	Stunting	HAZ: -2.1<br><br>Stunting Prevalence: 55.6%	HAZ: -1.4<br><br>Stunting Prevalence: 32.6%	3.21
Sub-Saharan Africa	Central African Republic, Chad, Democratic Republic of Congo, Ghana, Kenya (Mombasa), Kenya (Nyanza Province), Madagascar (South), Malawi, Mauritania, Nigeria, Sierra Leone, Somalia (Northeast), Somalia (Somaliland), Swaziland, Togo, Zimbabwe	Efevbera, 2017	Girl child marriage as a risk factor for early childhood development and stunting	Gray Literature	surveys from the fourth and fifth rounds of MICS, conducted between 2010 and 2014	Children aged 3-4 years	2010-2014	Child Characteristics, Maternal Characteristics, Region, Literacy, Birth Outcomes, Household SES	Child marriage (<18 years), geographic location (urban), mother completed primary school, multiple birth, gender (female), age (months), number of children ever-born to mother, mother's age at childbirth, mother completed secondary school, wealth quintile	Logistic Regression	Stunting			3.21
Latin America & Caribbean	Paraguay	Ervin, 2019	Closing the rural-urban gap in child malnutrition: Evidence from Paraguay, 1997-2012	Updated Search	Permanent Household Surveys (EPH) of 1997–98, 2000–01, 2005, and Household Income and Expenditure Survey (EIG) of 2011–12	Children 0-59 months	1997-2012	Household SES, Literacy, WASH, Unhealthy Household Environment, Health Services, Dietary Intake, Child Characterisitcs, Maternal Characteristics	Income, maternal education, paternal education, piped water, flush toilet, dirt floor, delayed vaccines, health insurance, born hospital, breastfeeding at birth, birth interval, birth order, caretaker under 20, sex, ethnicity, language	OLS Model<br><br>Linear Probability Model (LPM)<br><br>Oaxaca-Blinder Decomposition	HAZ<br><br>HAZ Inequality (urban-rural)<br><br>Stunting Inequality (urban-rural)	HAZ: -0.77<br><br>Stunting Prevalence: 14.4%	HAZ: -0.49<br><br>Stunting Prevalence: 11.28	3.21
South Asia	Bangladesh	Goyal, 2018	Exposure to ambient fine particulate air pollution in utero as a risk factor for child stunting in Bangladesh	Covidence	DHS 2004, 2007, 2011, 2014	Children under age 5	2004-2014	Maternal Characteristics, Child Characteristics, Birth Outcomes, Infectious Diseases & Morbidities, Literacy, WASH, Unhealthy Household Environment, Region, Household SES	Pm 2.5 exposure in utero, age of child (months), twin or triplet, female, first child, birth interval, had diarrhea in last two weeks, maternal height, maternal body mass index, age of mother at birth, maternal education, partner's education, improved water source in household, improved sanitation in household, cooking with solid fuel, urban residence, wealth index quintile	Poisson Regression	Stunting	Stunting Prevalence: 43%	Stunting Prevalence: 30%	3.29
East Asia & Pacific	Cambodia	Grefeuille, 2016	Persistent inequalities in child undernutrition in Cambodia from 2000 until today	Covidence	DHS 2000, 2005, 2010, 2014	Children 0-59 months	2000-2014	Child Characteristics, Literacy, Region, Household SES	Child sex, mother's education, residence, wealth quintile	Logistic Regression	Stunting Prevalence			3.21
Sub-Saharan Africa	Zambia	Hangoma, 2017	Explaining changes in child health inequality in the run up to the 2015 Millennium Development Goals (MDGs): The case of Zambia	Covidence	DHS 2007, 2014	Children aged 0-5 years	2007-2014	Health Services, Birth Outcomes, Child Characteristics, Dietary Intake, Literacy, Maternal Characteristics,	Delivery at a medical facility, birthweight, child's age, duration of breastfeeding, birth order, maternal education, maternal height, maternal weight, maternal age, occupation,	Multilevel Linear Probability Model<br><br>Concentration	Stunting Probability<br><br>Stunting Inequality	Stunting Prevalence: 45.6%	Stunting Prevalence: 40.0%	3.29

								Household SES, Region, WASH, Unhealthy Household Environment	type of residence, water, sanitation, household size, number of children below 5 years	Index<br><br>Decomposition of Concentration Index				
Sub-Saharan Africa	28 countries	Harttgen, 2013	Economic Growth and Child Undernutrition in sub-Saharan Africa	Review Reference Scoping	DHS between 1991-2009	Living children born five years prior to the survey	1991-2009	Macro Level: Economic Factors, Household SES, Region, Maternal Characteristics, Parental Characteristics, Literacy, Child Characteristics, Birth Outcomes	Log GDP per capita, asset index, urban, number of children survived, household head is male, age of household head, household head's education, age of mother at birth of child, mother currently pregnant, mother's BMI, mother's education, child is a boy, age of child, child is a twin	Pooled Logistic Regression	Stunting			3.21
South Asia	Bangladesh	Hasan, 2016	The role of maternal education in the 15-year trajectory of malnutrition in children under 5 years of age in Bangladesh	Covidence	DHS 1996-1997, 1999-2000, 2004, 2007, 2011	Children under 5 years of age	1996-2011	Literacy	Maternal Education	Log-Binomial Regression	Stunting	Stunting Prevalence: 60.0%	Stunting Prevalence: 41.2%	3.29
Sub-Saharan Africa	Ethiopia	Headey, 2014	An analysis of trends and determinants of child undernutrition in Ethiopia, 2000-2011	Gray Literature	DHS 2000, 2011	Preschool children	2000-2011	Household SES, Literacy, Health Services, Maternal Characteristics, WASH, Region, Child Characteristics	Asset index, maternal education, paternal education, 4+ antenatal visits, medical delivery, birth interval, birth order, open defecation, piped water, urban dummy, female child dummy, region	OLS Regression<br><br>LPM<br><br>Poisson Regression<br><br>Oaxaca-Blinder Decomposition	HAZ<br><br>Stunting	Stunting Prevalence: 55.7%	Stunting Prevalence: 43.4%	3.29
South Asia	Nepal	Headey, 2015a	Understanding the Rapid Reduction of Undernutrition in Nepal, 2001-2011	Covidence	DHS 2001, 2006, 2011	Children 0-59 months	2001-2011	Household SES, Literacy, Health Services, Maternal Characteristics, WASH, Women's Empowerment	Asset index, maternal education, paternal education, 4 or more antenatal care visits, iron during pregnancy, born in hospital, all vaccinations, preceding birth interval, open defecation, water source, women's empowerment, maternal height	Linear Regression<br><br>LPM<br><br>Oaxaca-Blinder Decomposition	HAZ<br><br>Stunting	HAZ: -2.17<br><br>Stunting Prevalence: 56.6%	HAZ: -1.66<br><br>Stunting Prevalence: 40.1%	3.29
South Asia	Bangladesh	Headey, 2015b	The other Asian enigma: explaining the rapid reduction of undernutrition in Bangladesh	Covidence	DHS 1997, 2000, 2004, 2007, 2011	Children 0-59 months	1997-2011	Household SES, Literacy, Health Services, WASH, Maternal Characteristics, Women's Empowerment	Asset index, maternal education, paternal education, all vaccines, prenatal doctor visit, born in medical facility, open defecation, piped water, birth order, birth interval, women's empowerment, maternal height	Linear Regression<br><br>LPM<br><br>Oaxaca-Blinder Decomposition	HAZ<br><br>Stunting	HAZ: -2.20<br><br>Stunting Prevalence: 58.5%	HAZ: -1.62<br><br>Stunting Prevalence: 40.2%	3.29
South Asia	Bangladesh<br>India<br>Nepal<br>Pakistan	Headey, 2016	Drivers of nutritional change in four South Asian countries: A dynamic observational analysis	Covidence	Bangladesh: DHS 1996/1997, 1999/2000, 2004, 2007, 2011<br>Nepal: DHS 1996, 2001, 2006, 2011<br>India: DHS 1992/1993, 2005/2006<br>Pakistan: 1991, 2013	Pre-school children aged 0 to 59 months	Bangladesh: 1997-2011<br>Nepal: 1996-2011<br>India: 1993-2006<br>Pakistan: 1991-2013	Household SES, Literacy, Health Services, WASH, Maternal Characteristics, Child Characteristics	Asset index, maternal education, paternal education, four or more antenatal visits, born in a medical facility, open defecation, water, birth interval, number of children, female child	OLS Regression<br><br>Decomposition	HAZ	HAZ:<br>Bangladesh: -2.2<br>India: -1.9<br>Nepal: -2.1<br>Pakistan: -2.0	HAZ:<br>Bangladesh: -1.6<br>India: -1.6<br>Nepal: -1.6<br>Pakistan: -1.7	3.29
South Asia, Sub-Saharan Africa	Bangladesh<br>Ethiopia<br>Odisha State, India<br>Nepal<br>Senegal<br>Zambia	Headey, 2017	Accounting for nutritional changes in six success stories: A regression-decomposition approach	Covidence	Bangladesh: DHS 1996/1997, 1999/2000, 2004, 2007, 2011, 2014<br>Ethiopia: DHS 2000, 2005, 2011<br>Odisha, India: DHS 1992/1993, 2005/2006<br>Nepal: DHS 1996, 2001, 2006, 2011<br>Senegal: DHS 1992/1993, 2005, 2010/2011<br>Zambia: DHS 2001/2002, 2007, 2013/2014	Bangladesh, Ethiopia, Senegal, Zambia: children 0-59 months<br>Nepal: 0-35 months<br>Odisha, India: 0-47 months	Bangladesh: 1997-2011<br>Ethiopia: 2000-2011<br>Odisha, India: 1993-2006<br>Nepal: 1996-2011<br>Senegal: 1993-2011<br>Zambia: 2002-2014	Household SES, Literacy, Health Services, WASH, Maternal Characteristics, Macro Level: Environmental and Community Context	Asset index, paternal education, maternal education, maternal height, sanitation, 4+ antenatal visits during pregnancy, children born in medical facility, total number of children ever born, households with bednets	Linear Regression<br><br>Decomposition	HAZ	HAZ:<br>Bangladesh: -2.28<br>Ethiopia: -2.16<br>Odisha State, India: -1.98<br>Nepal: -2.20<br>Senegal: -1.44<br>Zambia: -1.86	HAZ:<br>Bangladesh: -1.38<br>Ethiopia: -1.54<br>Odisha State, India: -1.65<br>Nepal: -1.62<br>Senegal: -1.14<br>Zambia: -1.48	3.29
East Asia & Pacific, Latin America & Caribbean, Middle East & North Africa, South Asia, Sub-Saharan Africa	49 low- and middle-income countries	Headey, 2018	Animal sourced foods (ASF) and child stunting	Gray Literature	phases 5 and 6 of DHS surveys between 2006-2014	Children 6 to 23 months	2006-2014	Dietary Intake, Macro Level: Economic Factors, Macro Level: Conflict/Insecurity, Household SES, Literacy, WASH, Women's Empowerment, Region, Paternal Characteristics, Health Services	ASF consumption, log own price, log cereal yields, log GDP per capita, log urbanization rate, conflict dummy, wealth tercile, maternal education, paternal education, open defecation, water, women's autonomy, breastfeeding, rural, father absent, hospital/clinic access	Pooled Bivariate Regression<br><br>Pooled LPM	Stunting			3.29
Sub-Saharan Africa	Kenya<br>Zambia	Hoffman, 2017	Temporal changes and determinants of childhood nutritional status in Kenya and Zambia	Covidence	Kenya: DHS 1998, 2003, 2009	Children birth to 4 years	Kenya: 1998-2009	Household SES, Unhealthy Household Environment, Region,	Wealth index, household members, setting, toilet type, electricity, mother	Multivariate Logistic Regression	Stunting	Stunting Prevalence:	Stunting Prevalence:	3.21

					Zambia: DHS 1996, 2002, 2007, 2014		Zambia: 1996-2014	WASH, Maternal Characteristics, Literacy, Child Characteristics	age, maternal education, child age, child sex,			Kenya: 7.5%<br>Zambia: 5.4%	Kenya: 8.1%<br>Zambia: 6.3%	
South Asia	Bangladesh	Huda, 2018	Social determinants of inequalities in child undernutrition in Bangladesh: A decomposition analysis	Covidence	DHS 2004, 2014	Children younger than 5 years of age	2004-2014	Maternal Characteristics, Region, Health Services, Literacy, Household SES, WASH,	Maternal age, maternal height, birth order, preceding birth interval, region, prenatal care from a medically trained provider, delivery at a health care facility, husband's education level, mother's education level, wealth quintile, improved sanitation, mother's employment status	Decomposition of Concentration Index	Stunting Inequality	Stunting Prevalence: 43%	Stunting Prevalence: 36%	3.21
Latin America & Caribbean	Peru	Huicho, 2017	Factors behind the success story of under-five stunting in Peru: A district ecological multilevel analysis	Covidence	DHS 2000, 2004, 2005, 2006, 2007, 2008, 2009, 2010, 2011, 2012	Children under 5 years	2000-2012	Macro Level: Economic Factors, Unhealthy Household Environment, Literacy, WASH, Fertility, Other Programs/Policies/Interventions, Health Services	Log GDP per capita, unmet basic needs, poverty line, Gini coefficient for income, urbanization, maternal education, improved water source, total fertility rate, cash transfer programme coverage, SIS utilization, per capita expenditure on child health activities, density of human resources, composite coverage index	Multilevel Mixed-Effects Regression	Stunting Prevalence			3.29
East Asia & Pacific	Cambodia	Ikeda, 2013	Determinants of reduced child stunting in Cambodia: analysis of pooled data from three demographic and health surveys	Covidence	DHS 2000, 2005, 2010	Children less than 5 years of age	2000-2010	Child Characteristics, Region, Birth Outcomes, Maternal Characteristics, Unhealthy Household Environment, WASH, Literacy	Child sex, age, type of residence, multiple birth %, breastfeeding at birth, birth interval, average number of household members, water, sanitation, incidence of diarrhoea, paternal education, maternal education, maternal use of tobacco, maternal height, maternal BMI, mother's age	Multivariate Hierachical Logistic Regression<br><br>Calculation of Relative Contribution	Stunting<br><br>Stunting Prevalence	Stunting Prevalence: 49.3%	Stunting Prevalence: 39.0%	3.21
Multi-Region	37 countries	Jahagirdar, 2017	The effect of paid maternity leave on early childhood growth in low-income and middle-income countries	Covidence	DHS 2000-2014	Children younger than 5 years	2000-2014	Maternal Characteristics, Unhealthy Household Environment, Literacy, Household SES, Other Programs/Policies/Interventions	Maternal age at pregnancy, maternal height, birth order, family size, birth month, maternal education, household wealth, legislated paid maternity leave	Pooled Linear Regression	HAZ			3.29
South Asia	India	Joe, 2016	Understanding the null-to-small association between increased macroeconomic growth and reducing child undernutrition in India: Role of development expenditures and poverty alleviation	Covidence	NFHS 1992/93, 2005/2006	Children under the age of 3 years	1992-2006	Macro Level: Economic Factors	PCNSDP, PCDE, HCR	Multilevel Logistic Regression	Stunting Prevalence			3.29
East Asia & Pacific	Mongolia	Joshi, 2017	Social and environmental determinants of child health in Mongolia across years of rapid economic growth: 2000-2010	Covidence	MICS 2000,2005, 2010	Children 0-59 months	2000-2010	Child Characteristics, Unhealthy Household Environment, Literacy, Household SES, WASH, Region	Sex, age, number of children under age 5 in the home, maternal education level, household economic status, water source, sanitation facility, place of residence, region of residence	Multivariate Multi-Level Logistic Mixed Modelling	Stunting	HAZ: -1.12<br><br>Stunting Prevalence: 25.5%	HAZ: -0.48<br><br>Stunting Prevalence: 13.3%	3.29
Sub-Saharan Africa	Kenya	Kabubo-Mariara, 2009	Determinants of children's nutritional status in Kenya: evidence from Demographic and Health Surveys	Covidence	DHS 1998, 2003	Children < 36 months	1998-2003	Child Characteristics, Birth Outcomes, Unhealthy Household Environment, Maternal Characteristics, Parental Characteristics, Literacy, Household SES, Health Services, WASH	Child's age, child is of multiple birth, male child dummy, share of women aged 15-49 years, household size, mother's age, mother's height, mother's education, head's years of education, age of household head, asset index, children received all vaccinations, mothers used professional prenatal care, expectant mothers received tetanus toxoid, women used professional birth care, women using modern contraception, households with piped water, households with traditional toilet	Survey Regression	HAZ<br><br>Stunting Probability	HAZ: -1.182<br><br>Stunting Probability: 0.33	HAZ: -1.184<br><br>Stunting Probability: 0.35	3.21
South Asia	Bangladesh	Khatun, 2019	Assessing the Intergenerational Linkage between Short Maternal Stature and Under-Five Stunting and Wasting in Bangladesh	Updated Search	DHS 2004, 2007, 2011, 2014	Children aged 0–59 months born to mothers aged 15–49 years	2004-2014	Maternal Characteristics, Literacy, Household SES, Child Characteristics, Region	Maternal height, Maternal age, Maternal education level, Mother’s occupation, Child age, Birth order, Birth interval, Sex of the child, Wealth quintile, Father’s education, Location of residence, Year of survey	Multivariable Poisson Regression	Stunting	Stunting Prevalence: 27.3%	Stunting Prevalence: 23.6%	3.07
East Asia & Pacific	Vietnam	Kien, 2016	Trends in socioeconomic inequalities in child malnutrition in Vietnam: findings from the Multiple Indicator Cluster Surveys, 2000-2011	Covidence	MICS 2000, 2011	Under-five children	2000-2011	Child Characteristics, Region, Literacy, Household SES	Child's age, sex of child, area, ethnicity, mother's education, socioeconomic status	Multivariable Logistic Regression<br><br>Decomposition of Concentration Index	Stunting<br><br>Stunting Inequality	Stunting Prevalence: 36.7%	Stunting Prevalence: 22.7%	3.21

Sub-Saharan Africa	Somalia	Kinyoki, 2015	Predictors of the risk of malnutrition among children under the age of 5 years in Somalia	Covidence	FSNAU household cross-sectional nutritional surveys from 2007-2010	Children 6-59 months	2007-2010	Dietary Intake, Health Services, Infectious Diseases & Morbidities, Child Characteristics, Unhealthy Household Environment, Female Empowerment, Maternal Characteristics, Macro Level: Environmental and Community Context	Vitamin A Supplementation, Measles Vaccination, Polio Vaccination, Diarrhea, ARI, Febrile Illness, Suspected Measles, Sex Of The Child, Age Of The Child, Household Size, Number Of Under-5s, Female Household Head, Age Of The Mother, MUAC Of Mother, Food And Nutrition Consumption, Season, Distance To Water, EVI, Rainfall, Temperature, Urbanization	Spatial-Temporal Bayesian Logistic Regression	Stunting Prevalence			3.29
East Asia & Pacific	Cambodia	Kov, 2013	Growing Taller Among Toilets: Evidence from Changes in Sanitation and Child Height in Cambodia, 2005 – 2010	Review Reference Scoping	DHS 2005, 2010	Children under 5 years old	2005-2010	WASH, Unhealthy Household Environment, Region, Maternal Characteristics	Sanitation, electrification, type of residence, mother's height, mother's BMI	Non-Parametric Local Regression<br><br>OLS Regression<br><br>Fixed-Effects Regression<br><br>Blinder-Oaxaca Decomposition	Height	HAZ: -1.77	HAZ: -1.64	3.21
South Asia	Bangladesh<br>India<br>Nepal<br>Pakistan	Krishna, 2017	Trends in inequalities in child stunting in South Asia	Covidence	DHS 1991-2014	Children ages 6–23 months	1991-2014	Dietary Intake, Literacy, Household SES	Dietary diversity score, mother's education, wealth quintile	Logistic Regression	Stunting Prevalence	Stunting Prevalence:<br>Bangladesh: 52.7%<br>India: 51.3%<br>Nepal: 53.4%<br>Pakistan: 45.2%	Stunting Prevalence:<br>Bangladesh: 32.0%<br>India: 43.5%<br>Nepal: 28.3%<br>Pakistan: 39.7%	3.29
Sub-Saharan Africa	Tanzania	Lovo, 2019	Crop Diversification and Child Health: Empirical Evidence From Tanzania	Updated Search	Tanzania National Panel Survey (TZNPS) 2008/ 2009, 2010/2011, and 2012/2013	children aged 0–10 years	2008-2013	Literacy, Child Characteristics, Unhealthy Household Environment, Macro Level: Economic Factors, Household SES	Crop diversification, Child education, Child age, Household number of children, Elderly members, Land size, Household consumption, Total revenues, Livestock, Off-farm employment	Baseline Regression<br><br>IV and IIV Approach	HAZ			3.00
Sub-Saharan Africa	Kenya	Masibo, 2012	Trends and determinants of undernutrition among young Kenyan children: Kenya Demographic and Health Survey; 1993, 1998, 2003 and 2008-2009	Covidence	DHS 1993,1998, 2003, 2008-2009	Children 0-59 months	1993-2009	Region, Household SES, WASH, Maternal Characteristics, Literacy, Unhealthy Household Environment, Child Characteristics, Birth Outcomes, Infectious Diseases & Morbidities	Residence, province, wealth index, source of drinking water, toilet facilities, mother's age, martial status, maternal education, BMI, work status, number of children, 5 years old living with mother, child sex, child age, birth order, size at birth, incidence of diarrhoea, fever, cough	Binary Logistic Regression	Stunting	Stunting Prevalence: 39.9%	Stunting Prevalence: 35.3%	3.21
Sub-Saharan Africa	Kenya	Matanda, 2014	Child undernutrition in Kenya: Trend analyses from 1993 to 2008-09	Covidence	DHS 1993, 1998, 2003, 2008-09	Children aged 0-35 months	1993-2009	Child Characteristics, Region, Literacy, Household SES	Child's sex, child's age, province, residence, maternal education, wealth index	Logistic Regression	Stunting	Stunting Prevalence: 39.5%	Stunting Prevalence: 36.5%	3.29
Multi-Region	85 developing countries	Milman, 2005	Differential improvement among countries in child stunting is associated with long-term development and specific interventions	Covidence	FAO, UNDP, World Bank Development Indicators, UNICEF	Children <5 y old	1971-2001	Health Services, WASH, Dietary Intake, Literacy, Macro Level: Environmental and Community Context, Macro Level: Political Context, Macro Level: Economic Factors	Immunization rate, safe water, daily energy supply, female literacy rate, HIV/AIDS, urban population, civil liberties and political rights, log per capita GNP	Pooled Multiple Linear Regression	Stunting			3.21
South Asia	Bangladesh	Mohsena, 2017	Socioeconomic and Demographic Variation in Nutritional Status of under-Five Bangladeshi Children and Trend over the Twelve-Year Period 1996-2007	Covidence	DHS 1996/1997, 1999/2000, 2004, 2007	Children aged 0-5 years	1996-2007	Region, Household SES, Literacy, Unhealthy Household Environment, WASH	Region, residence, occupation of fathers, occupation of mothers, education of fathers, education of mothers, possession score, house type, water, toilet, number of household members	General Linear Model<br><br>Sequential Linear and Binary Logistic Regression	HAZ<br><br>Stunting	Stunting Prevalence: 57%	Stunting Prevalence: 41%	3.21
Latin America & Caribbean	Brazil	Monteiro, 2009	Causes for the decline in child undernutrition in Brazil, 1996-2007	Covidence	DHS 1996, 2006/2007	Children under five years of age	1996-2007	Household SES, Literacy, Health Services, WASH	Purchasing power classes, maternal schooling, health care, sanitation	Poisson Multiple Regression	Stunting Prevalence<br><br>Stunting Probability	Stunting Prevalence: 13.5%	Stunting Prevalence: 6.8%	3.21
Latin America & Caribbean	Brazil	Monteiro, 2010	Narrowing socioeconomic inequality in child stunting: the Brazilian experience, 1974--2007	Covidence	Estudo Nacional de Despesa Familiar [National Study on Family Expenditures] 1974–75; Pesquisa Nacional de Saúde e Nutrição [National Health and Nutrition Survey] 1989; Demographic and Health Surveys 1996 and 2006–07	Children aged 0–59 months	1974-2007	Household SES, Literacy, Health Services, WASH, Maternal Characteristics	Per capita income, household assets, maternal schooling, prenatal care, water and sanitation services, birth order, birth interval, modern contraceptive use	Slope Index of Inequality	Stunting Inequality<br><br>Concentrati on Index<br><br>Erreygers Concentrati on Index	Stunting Prevalence: 37.1%	Stunting Prevalence: 7.1%	3.21

South Asia	Nepal	Nepali, 2019	Trends and inequalities in stunting in Nepal: a secondary data analysis of four Nepal demographic health surveys from 2001 to 2016	Updated Search	DHS 2001, 2006, 2011, 2016	Children under 5 years old	2001-2016	Region, Literacy, Household SES, Macro Level: Environmental and Community Context	Type of place of residence, Mother's education, Wealth quintiles, Development regions, Ecological zones	Logistic Regression	Stunting	Stunting Prevalence: 51%	Stunting Prevalence: 35.8%	3.21
South Asia	India	Nie, 2019	Changes in Child Nutrition in India: A Decomposition Approach	Updated Search	Indian Human Development Survey 2004-2005, 2011-2012	Children aged 0–5 years	2004-2012	Women's Empowerment, Maternal Characteristics, Macro Level: Economic Factors, WASH, Region	Mother's decision-making autonomy, Mother's mobility autonomy, Mother's characteristics, Economic situation, Hygiene, Region	Blinder-Oaxaca Decomposition<br><br>Re-centered Influence Function Regression (RIFR) Decomposition<br><br>Fairlie's (1999) Non-Linear Decomposition	Stunting Inequality	HAZ: -1.3931<br><br>Stunting Prevalence: 42.94%	HAZ: -1.1682<br><br>Stunting Prevalence: 35.77%	3.07
Sub-Saharan Africa	Malawi	Ntenda, 2017	Analysis of individual-level and community-level effects on childhood undernutrition in Malawi	Covidence	DHS 2004, 2010	Children under the age of 5 years	2004-2010	Child Characteristics, Birth Outcomes, Infectious Diseases & Morbidities, Maternal Characteristics, Literacy, Unhealthy Household Environment, Household SES, Region, Macro Level: Environmental and Community Context, Health Services	Child's sex, child's age, size of the child at birth, fever in the last weeks, recent diarrheal disease, acute respiratory infection, type of birth, maternal HIV status, mother's age, mother's educational level, mother's BMI, number of under 5 in the household, household wealth, place of residence, geographical region, community wealth, community female education, community HIV status, community prenatal care, community skilled delivery, distance to health facility	Generalized Estimating Equations Logistic Regression	Stunting			3.29
East Asia & Pacific	Vietnam	O'Donnell, 2009	Growing richer and taller: Explaining change in the distribution of child nutritional status during Vietnam's economic boom	Review Reference Scoping	Vietnam Living Standards Survey 1993, 1998	Children 0-10 years	1993-1998	Macro Level: Economic Factors, WASH, Child Characteristics, Maternal Characteristics, Paternal Characteristics, Literacy, Parental Characteristics, Unhealthy Household Environment, Region	Log household consumption per capita, log commune mean household consumption per capita, safe drinking water, sanitary toilet, child's sex, mother's age, mother's height, father's height, mother's education, father's education, mother dead or absent, father dead or absent, household size, kids as proportion of household size, ethnic minority, urban	Quantile Regression<br><br>Machado and Mata (2005) Decomposition	Stunting Inequality	Stunting Proportion: 0.5255<br><br>HAZ: -2.0334	Stunting Proportion: 0.3745<br><br>HAZ: -1.6248	3.14
Sub-Saharan	South Africa	Otterbach, 2019	Exploring spatial differences in the risk of child stunting: evidence from a South African national panel survey	Updated Search	South African National Income Dynamics Study (NIDS) 2008, 2010, 2012 and 2014	Children 0-19 years, stratified 0-59 months in analysis	2008-2014	Household SES, Region, Child Characteristics, Women's Empowerment, Literacy, WASH, Unhealthy Household Environment	Ln real equivalized hh income, subsistence farming, geographical classification, child's sex, hh head is female, hh head educational level, number of employed persons in hh, having access to flush toilet, having access to tap water, removal of refuse and rubbish, hh has electricity, population group	Random-Effects Logistic Regression	Stunting	Stunting Prevelance: 16.7%	Stunting Prevalence: 12.4%	3.14
East Asia & Pacific	Cambodia	Pierce, 2019	Increasing health facility deliveries in Cambodia and its influence on child health	Updated Search	DHS 2010, 2014	Women of child-bearing age and children	2010-2014	Literacy, Household SES, Region	Maternal education, Household wealth, Urban residence, Year	Logistic Regression<br><br>Linear Regression<br><br>Cox Regression<br><br>Propensity Score Matching	Health Facility Utilization<br><br>HAZ<br><br>Socioeconomic Inequality in HAZ			3
Multi-Region	23 low- and middle-income countries (LMICs)	Ponce, 2017	The association of minimum wage change on child nutritional status in LMICs: A quasi-experimental multi-country study	Covidence	DHS 2003-2012 Maternal and Child Health Equity (MACHEquity) research program	Children under 5 years	2003-2012	Macro Level: Economic Factors, Health Services, Fertility, Unhealthy Household Environment, Maternal Characteristics, Literacy, Household SES, Region, Child Characteristics	Log per capita GDP, log per capita health expenditures, public (ppp), % share of wage and salary workers, fertility, % urban population, water access, internet, mobile, maternal age, maternal height, mother's education, mother's marital status, number of children under age 5 years living in household, wealth quintile, rural, child age, sex, birth order	Pooled Generalized Difference-in-Difference Model				3.29
Sub-Saharan	Nigeria	Rabassa, 2012	Weather and Child Health in Rural Nigeria	Gray Literature	DHS 2003, 2008	Children 0 to 35 months	2003-2008	Macro Level: Environmental and Community Context, Health Services, Child Characteristics	Rainfall shocks, health center, child's age, child's sex	OLS Regression	HAZ			3.14

South Asia	Bangladesh	Rabbani, 2016	Trends and determinants of inequities in childhood stunting in Bangladesh from 1996/7 to 2014	Covidence	DHS 1996/1997-2014; Child Nutrition Surveys in 1986, 1990, 1992, 1996, 2000, and 2005; Household Food Security and Nutrition Assessment data 2009;Bangladesh Food Security and Nutritional Surveillance Project surveys from 2010-2013	Children under 5	1996-2014	Child Characteristics, Maternal Characteristics, Health Services, Dietary Intake, Literacy, Household SES	Child's age, birth order, antenatal visit to doctor, delivery at health facility, early initiation of breastfeeding, maternal schooling (years), paternal schooling (years), maternal CED (chronic energy deficiency), maternal short stature, wealth index	Equity Analysis<br>Oaxaca-Blinder Decomposition of Concentration Index	Stunting Inequality	Stunting Rate: 60.0%<br>HAZ: -2.33	Stunting Rate: 36.1%<br>HAZ: -1.54	3.07
South Asia	Sri Lanka	Rannan-Eliya, 2013	Trends and determinants of childhood stunting and underweight in Sri Lanka	Covidence	DHS 1987, 1993, 2000, 2006-07 Nutrition and Food Security Survey (NFSS) 2009	Children age 0-59 months (DHS 2006/07, NFSS 2009), 3-59 months (DHS 1993 and 2000) and 3-36 months (DHS 1987)	1987-2009	Child Characteristics, Birth Outcomes, Maternal Characteristics, Literacy, Dietary Intake, Household SES, Unhealthy Household Environment, WASH, Macro Level: Environmental and Community Context	Child age, child sex, first child, birth weight, maternal age, maternal height, maternal BMI, mother's education, mother's ethnicity, breastfeeding, dietary diversity, iron rich diet, minimum meal frequency, dairy products for youngest child, number of foods for youngest child, solid/semisolid food by 9 months, household wealth quintile, number of children in household (age<5), improved sanitary conditions, altitude	Concentration Index<br>OLS Regression	Stunting Inequality<br>HAZ	Stunting Prevalence: 31.1%	Stunting Prevalence: 18.3%	3.21
Middle East & North Africa	Egypt	Rashad, 2015	Does economic growth reduce child malnutrition in Egypt? New evidence from national demographic and health survey	Covidence	DHS 1992, 2000, 2003, 2005, and 2008	Children 0-59 months	1992-2008	Macro Level: Economic Factors, Maternal Characteristics, Literacy, Household SES, Health Services, Paternal Characteristics, Child Characteristics, Birth Outcomes, Household SES, WASH	Log GDP, Gini index, mother's age, mother's education, mother's occupation, mother's nutrition, pregnancy status, healthcare during pregnancy, father's education level, father's occupation, child's sex, current age of child, child is twin, birth interval, wealth index, access to clean water, sanitation facilities	Multilevel Logistic Regression	Stunting	Stunting Prevalence: 25%	Stunting Prevalence: 28.9%	3.14
Multi-Region	Kenya, Madagascar, Malawi, Rwanda, Tanzania, Uganda, Zambia, Zimbabwe, Benin, Burkina Faso, Cameroon, Gabon, Ghana, Mali, Egypt, Jordan, Bangladesh, Nepal, Cambodia, Bolivia, Colombia, Dominican Republic, Haiti, Peru	Restrepo-Mendez, 2015	Time trends in socio-economic inequalities in stunting prevalence: analyses of repeated national surveys	Covidence	DHS and MICS 1993-2012	Children <5 years of age	1993-2012	Household SES	Wealth Index	SII<br>Concentration Index	Stunting Inequality			3.14
Multi-Region	Burkina Faso, Cote d'Ivoire, Democratic Republic of Congo, Nepal, Rwanda, Uganda, Cambodia, Chad, Ghana, Mali, Mozambique, Cameroon, Egypt, Madagascar, Malawi, Niger, Nigeria, Tanzania, Turkey, Zambia, Bangladesh, Peru, Ethiopia, Kenya, India	Rieger, 2014	Temporal stability of child growth associations in Demographic and Health Surveys in 25 countries	Updated Search	DHS 1991-2014	Children aged 0–59 months	1991-2014	Child Characteristics, Maternal Characteristics, Fertility, Literacy, Unhealthy Household Environment, Macro Level: Environmental and Community Context, Infectious Diseases & Morbidities, Region	Child sex, Birth order, Maternal Height, Age at birth, Age at first marriage, Fertility, Maternal education, Paternal education, Household size, Community and infrastructure, Mortality of under-5 children in previous five years in the primary sampling unit, Household access to electricity, Rural area of residence	Linear Regression<br>Parsimonious Model	HAZ			3.07
South Asia	Bangladesh	Saha, 2019	Trends, prevalence and determinants of childhood chronic undernutrition in regional divisions of Bangladesh: Evidence from demographic health surveys, 2011 and 2014	Updated Search	DHS 2011, 2014	children aged 6–59 months	2011-2014	Region, Child Characteristics, Maternal Characteristics, Literacy, Household SES, Health Services	Birth regions, Type of residence, Survey year, Age of child (months), Gender of child, Religion, Birth order, Mother's age at birth, Mother's BMI, Mother's height, Schooling years of Mother, Schooling years of Father, Household wealth quintiles, Distance to nearest health clinics (kilo.)	Bivariate Regression<br>Logistic Regression	Stunting Prevalence			3.14
Latin America & Caribbean	Peru	Sobrino, 2017	Birth interval and stunting in children under five years of age in Peru (1996–2014)	Covidence	DHS 1996, 2000, 2005, 2010, 2014	Children <5 years of age	1996-2014	Maternal Characteristics, Literacy, Region, Household SES	Birth interval, birth order, gender, maternal age, mother instruction, residence, natural region, wealth	Logistic Regression	Stunting			3.29
Multi-Region	65 developing countries	Spears, 2013	How Much International Variation in Child Height Can Sanitation Explain?	Gray Literature	DHS 1990-2010	Children under 3 and children under 5	1990-2010	Macro Level: Economic Factors, Maternal Characteristics, Macro Level: Environmental and Community Context, Unhealthy Household	GDP, women's height, population density, village level open defecation, female literacy, nearby water, calorie deficit, electrification, urban/rural, household open defecation	Descriptive Regression<br>Econometric Decomposition	Stunting			3.21

								Environment, Literacy, Dietary Intake, Region, WASH						
South Asia	Nepal	Smith, 2019	Multilevel analysis of individual, household, and community factors influencing child growth in Nepal	Updated Search	DHS 2006, 2011	Children aged 0 to 59 months with complete anthropometric measurements	2006-2011	Child Characteristics, Birth Outcomes, Dietary Intake, Infectious Diseases & Morbidities, Literacy, Maternal Characteristics, Household SES, WASH, Macro Level: Environmental and Community Context, Food Security, Macro Level: Economic Factors, Health Services	Child Age, Gender, Twin, Still breastfeeding, Months breastfeeding, Fever in the last two weeks, Diarrhea in the last two weeks, Mother's education, Access to handwashing, Mother's BMI, Mother's age at birth, Wealth index, Water purification, Year, Altitude, Mother is a Dalit, Biomass Usage, Food shortage , Gender equity, Marginal, Commercial, Hospital distance, ODF free	Multilevel Regression	HAZ			3.07
Latin America & Caribbean	Peru	Stifel, 2006	The 'Glass of Milk' Subsidy Program and Malnutrition in Peru	Review Reference Scoping	DHS 1996, 2000	Children 0–59 months of age	1996-2000	Macro Level: Economic Factors, Child Characteristics, Birth Outcomes, Unhealthy Household Environment, Parental Characteristics, Maternal Characteristics, Literacy, WASH, Region	Per capita Vaso de Leche district expenditures, log per capita household expenditures (predicted), sex, multiple birth, birth order, child's age, household composition, number of household members, household head gender, ethnicity, mother's age, mother's height, mother's education, father's education, house floor condition, piped drinking water, flush toilet, urban/rural	Ordinary Linear Two-Stage Least Squares Regression	HAZ	Stunting Prevalence: 26.0%<br><br>HAZ: -1.07	Stunting Prevalence: 25.8%<br><br>HAZ: -1.18	3.14
South Asia	India	Subramanyam, 2010	Socioeconomic inequalities in childhood undernutrition in India: Analyzing trends between 1992 and 2005	Covidence	National Family Health Survey of India 1992, 1998, 2005	Children aged less than three years	1992-2006	Child Characteristics, Household SES, Literacy, Region	Gender, wealth quintile, caste, maternal education, type of residence	Multivariable Logistic Regression	Probability of Stunting	Stunting Prevalence: 52.43%	Stunting Prevalence: 44.73%	3.29
South Asia	India	Subramanyam, 2011	Is economic growth associated with reduction in child undernutrition in India?	Covidence	NFHS 1992-93, 1998-99, 2005-06	Children between 0 and 35 months in age and alive at the time of survey	1992-2006	Child Characteristics, Maternal Characteristics, Literacy, Household SES, Region	Child's age, child's gender, birth order, maternal age, maternal education, paternal education, marital status, household wealth, caste, religion, residence	Multilevel Logistic Regression	Stunting	Stunting Prevalence: 52.4%	Stunting Prevalence: 45.9%	3.29
East Asia & Pacific	Cambodia	Sunil, 2015	Decomposition of childhood malnutrition in Cambodia	Covidence	DHS 2000, 2005	Children 0 to 59 months	2000-2005	Child Characteristics, Maternal Characteristics, Health Services, Dietary Intake, Literacy, Household SES, WASH, Region	Child's age, sex of child, mother's age, underweight mother, prenatal care, delivery care, breastfeeding, vaccination, mother's education, mother's occupation, father's education, father's occupation, household wealth index, safe drinking water, toilet facility, type of residence	Bivariate Analysis<br><br>Logistic Regression<br><br>Decomposition	Stunting	Stunting Prevalence: 50.10%	Stunting Prevalence: 42.26%	3.21
North America	United States	Surkan, 2012	Impact of maternal depressive symptoms on growth of preschool-and school-aged children	Covidence	Early Childhood Longitudinal Study, Birth Cohort 2001-2007	Longitudinal time points when children were 9 months, 4 years, and 5 to 6 years	2001-2007	Maternal Characteristics	Maternal depressive symptons	Multivariable Logistic Regression	≤ 10% Height-for-Age			3.29
Latin America & Caribbean	Peru	Urke, 2014	Trends in stunting and overweight in Peruvian pre-schoolers from 1991 to 2011: findings from the Demographic and Health Surveys	Covidence	DHS 1991-92, 1996, 2000, 2007-08, 2011	Children 0 to 59 months	1991-2011	Region, Literacy	Residence, geographic region, maternal education	Logistic Regression	Stunting Prevalence	Stunting Prevalence: 37.4%	Stunting Prevalence: 19.3%	3.21
East Asia & Pacific	Vietnam	Wagstaff, 2003	On Decomposing the Causes of Health Sector Inequalities with an Application to Malnutrition Inequalities in Vietnam	Gray Literature	Vietnam Living Standards Surveys (VLSS) 1993, 1998	Children under the age of ten	1993-1988	Child Characteristics, Macro Level: Economic Factors, WASH, Literacy	Child's age, child sex, household consumption, safe drinking water, satisfactory sanitation, years schooling household head, years schooling mother	Linear Regression<br><br>Inequality Decomposition<br><br>Oaxaca-type Decomposition<br><br>Total Differential Decomposition	Stunting Inequality			3.14
Sub-Saharan Africa	Ethiopia	Woodruff, 2017	Determinants of stunting reduction in Ethiopia 2000 - 2011	Covidence	DHS 2000, 2005, 2011	Children 0–59 months of age	2000-2011	Maternal Characteristics, Birth Outcomes, Infectious Diseases & Morbidities, Dietary Intake, Literacy, Unhealthy Household Environment, Parental	Birth order, estimated size at birth, incidence of diarrhea, fever, non-human milk consumption, mother's height, mother's BMI, mother's educational level, number of mother's living children, number of children under 5 in household, age of	Bivariate Linear Regression<br><br>Multivariate Linear Regression	HAZ	HAZ: -2.08<br><br>Stunting Prevalence: 57.7%	HAZ: -1.61<br><br>Stunting Prevalence: 44.3%	3.21

								Characteristics, Household SES, WASH	household head, dependency ratio, household wealth index, household open defecation	Trends Analysis				
Sub-Saharan Africa	Guinea	Woodruff, 2018	Determinants of stunting, wasting, and anemia in Guinean preschool-age children: An analysis of DHS data from 1999, 2005, and 2012	Gray Literature	DHS 1999, 2005, 2012	Children younger than 5 years	1999-2012	Birth Outcomes, Maternal Characteristics, Infectious Diseases & Morbidities, Household SES, Dietary Intake, Unhealthy Household Environment, WASH, Region, Literacy, Health Services	Estimated birth size, child is twin, maternal vitamin a supplementation, child anemia, mother wants more children, mother's height, mother's BMI, household wealth quintile, child's sex, child currently breastfeeding, number of children <5 years in household, maternal anemia, household dependency ratio, household has safe water source, household disposes of child stool appropriately, place of residence, mother's partner had some formal education, incidence of fever, score for justification for wife beating, mother received skilled antenatal care	Bivariate Analysis<br><br>Logistic Regression	Stunting			3.29
South Asia	Bangladesh	World Bank.Operations Evaluation Department., 2005	Maintaining Momentum to 2015? An Impact Evaluation of Interventions to Improve Maternal and Child Health and Nutrition in Bangladesh	Gray Literature	DHS, Contraceptive Prevalence Survey (CPS), Bangladesh Fertility Survey (BFS), Bangladesh Retrospective Survey of Fertility and Mortality (BRSFM), World Fertility Survey Bangladesh (WFS), the Helen Keller International Health and Nutritional Survey (HNS), Health and Demographic Survey (HDS), and Maternal Mortality Survey	Children aged less than 5 years old	1996-2000	Household SES, Unhealthy Household Environment, WASH, Literacy, Maternal Characteristics, Women's Empowerment, Child Characteristics, Region, Health Services, Food Security, Birth Outcomes, Other Programs/Policies/Interventions	Wealth, finished floor, electricity, sanitary toilet, piped water, mother's education, adult/child ratio, mother age, female head, mother mobility, mother agency, mother height, child sex, birth order, pregnancy interval < 24 months, child's age, rural, birth month, division, measles vaccination, food deficit, low birth weight, Bangladesh Integrated Nutrition Project	OLS Regression<br><br>Two-Step Regression<br><br>Fixed-Effects Regression<br><br>Decomposition	HAZ			3.21
Sub-Saharan Africa	Multi-country	World Bank, 2017	Stunting Reduction in Sub-Saharan Africa	Gray Literature	Joint Malnutrition Estimates (JME)World Bank's World Development Indicators (WDI)<br>WHO<br>Demographic and Health Surveys (DHS)	Children under age five	1990-2015	Macro Level: Economic Factors, Food Security, Macro Level: Environmental and Community Context, WASH, Macro Level: Conflict/Insecurity, Macro Level: Political Context	GDP per capita, income elasticities, past economic recession, diversification of GDP, trade, lagged public health expenditure, lagged public education expenditure, Gini coefficient, female/male life expectancy ratio, food supply, people practicing open defecation, improved sanitation facilities, improved water source, control of corruption, government effectiveness, political stability and absence of violence, regulatory quality, rule of law, state fragility index, political violence episodes, number of conflicts	Quantile Regression<br><br>Augmented Mixed-Effects Regression<br><br>Panel Regression<br><br>Pooled OLS<br><br>IV Two-Stage Least Squares (2SLS) Regression	ln(Stunting)	Stunting Prevalence: 49%	Stunting Prevalence: 35%	3.21
East Asia & Pacific	China	Wu, 2015	The relationship between socioeconomic development and malnutrition in children younger than 5 years in China during the period 1990 to 2010	Covidence	National Bureau of Statistics of China (NBSC)<br>China Food and Nutrition Surveillance System (CFNSS)<br>National Nutrition Survey (NNS)	Children younger than 5 years	1990-2010	Macro Level: Economic Factors, Literacy, Unhealthy Household Environment, Food Security	GDP per capita, Engel coefficient, illiteracy, family size, education, cultural and recreation services consumption per person, food consumption per person	OLS Regression	Stunting Prevalence			3.21
Sub-Saharan Africa	Uganda	Yang, 2018	Trends and determinants of stunting among under-5s: evidence from the 1995, 2001, 2006 and 2011 Uganda Demographic and Health Surveys	Updated Search	DHS 1995, 2001, 2006, 2011	Children 0-59 months	1995-2011	Region, WASH, Unhealthy Household Environment, Maternal Characteristics, Literacy, Child Characteristics, Birth Outcomes, Infectious Diseases & Morbidities	Region, residence, source of drinking water, toilet facility, number of household members. Number of children under 5 in household, maternal age, BMI/pregnancy status, marital status, highest educational level, work status, child sex, child age, low birth size, birth order, birth interval, incidence of fever, diarrhoea, cough	Univariable and Bivariable Analysis<br><br>Multivariable Linear and Logistic Regression	Stunting	Stunting Prevalence: 44.8%	Stunting Prevalence: 33.2%	3.29
East Asia & Pacific	Cambodia	Zanello, 2016	What explains Cambodia's success in reducing child stunting-2000-2014?	Covidence	DHS 2000, 2004, 2010, 2014	Children under the age of five years	2000-2014	Child Characteristics, Health Services, Dietary Intake, Unhealthy Household Environment, Household SES, Literacy, Maternal Characteristics, WASH, Region	Child's age, gender, birth in hospital, breastfeeding after birth, vaccinations, prenatal visits, dependency ratio, mother's occupational status, mother's education, father's education, mother's BMI, asset index, improved sanitation, improved drinking water, regional characteristics	ANOVA<br><br>Quantile Regression-based Decomposition	HAZ	HAZ: -1.897<br><br>Stunting Prevalence: 51.5%	HAZ: -1.433<br><br>Stunting Prevalence: 33.9%	3.21

List of Included Studies

*** indicates national-level regression-decomposition studies**

Adesugba M, Edeh H, Mavrotas G. Child nutritional status, welfare and health in Nigerian households. IFPRI - Discussion Papers; 2018 (1776):iv + 25 pp many ref. Washington: International Food Policy Research Institute; 2018.

Adhikari RP, Shrestha ML, Acharya A, Upadhaya N. Determinants of stunting among children aged 0–59 months in Nepal: findings from Nepal Demographic and health Survey, 2006, 2011, and 2016. BMC Nutrition. 2019;5(1).

Adjaye-Gbewonyo K, Vollmer S, Avendano M, Harttgen K. Agricultural trade policies and child nutrition in low- and middle-income countries: a cross-national analysis. Global health. 2019;15(1):21.

Akombi BJ, Agho KE, Renzaho AM, Hall JJ, Merom DR. Trends in socioeconomic inequalities in child undernutrition: Evidence from Nigeria Demographic and Health Survey (2003 - 2013). PLoS One. 2019;14(2):e0211883.

Alfani FD, Andrew; Fisker, Peter; Molini, Vasco. Vulnerability to Malnutrition in the West African Sahel. Washington, DC.: World Bank Group; 2015. Contract No.: 7171.

Almasi A, Saeidi S, Zangeneh A, Ziapour A, Choobtashani M, Saeidi F, et al. Investigation of some Factors Affecting Stunting and Wasting among the Under-Five Children in Eastern Mediterranean Region. International Journal of Pediatrics-Mashhad. 2019;7(7):9759-72.

Amaral MM, Herrin WE, Gulere GB. Using the Uganda National Panel Survey to analyze the effect of staple food consumption on undernourishment in Ugandan children. BMC Public Health. 2017;18(1):32.

Amarante V, Figueroa N, Ullman H. Inequalities in the reduction of child stunting over time in Latin America: evidence from the DHS 2000-2010. Oxford Development Studies. 2018;46(4):519-35.

Ambel AA, Andrews C, Bakilana AM, Foster EM, Khan Q, Wang H. Examining changes in maternal and child health inequalities in Ethiopia. Int J Equity Health. 2017;16(1):152.

Angdembe MR, Dulal BP, Bhattarai K, Karn S. Trends and predictors of inequality in childhood stunting in Nepal from 1996 to 2016. Int J Equity Health. 2019;18(1):42.

Bagmar SH, Khudri M. Tracking Changes and Identifying Determinants of Child Malnutrition Status over the Past Decade in Bangladesh. Pakistan Journal of Nutrition. 2015;14(12):964-71.

Ballew C, Khan LK, Kaufmann R, Mokdad A, Miller DT, Gunter EW. Blood lead concentration and children's anthropometric dimensions in the Third National Health and Nutrition Examination Survey (NHANES III), 1988-1994. J Pediatr. 1999;134(5):623-30.

Barankanira E, Molinari N, Msellati P, Laurent C, Bork KA. Stunting among children under 3 years of age in Cote d'Ivoire: spatial and temporal variations between 1994 and 2011. Public health nutrition. 2017;20(9):1627-39.

Biadgilign S, Ayenew HY, Shumetie A, Chitekwe S, Tolla A, Haile D, et al. Good governance, public health expenditures, urbanization and child undernutrition Nexus in Ethiopia: an ecological analysis. BMC Health Serv Res. 2019;19(1):40.

Biadgilign S, Shumetie A, Yesigat H. Does Economic Growth Reduce Childhood Undernutrition in Ethiopia? PLoS One. 2016;11(8):e0160050.

Boccanfuso D, Bruce O. A New Avenue for Understanding the Nutritional Status of Children in Guinea. European Journal of Development Research. 2013;25(5):714-36.*

Buisman LR, Van de Poel E, O'Donnell O, van Doorslaer EKA. What explains the fall in child stunting in Sub-Saharan Africa? SSM Popul Health. 2019;8:100384.*

Christiaensen L, Alderman H. Child Malnutrition in Ethiopia: Can Maternal Knowledge Augment the Role of Income? Economic Development and Cultural Change. 2004;52(2):287-312.

Corsi DJ, Gaffey MF, Bassani DG, Subramanian SV. No Female Disadvantage in Anthropometric Status among Children in India: Analysis of the 1992-1993 and 2005-2006 Indian National Family Health Surveys. Journal of South Asian Development. 2015;10(2):119-47.

Cunningham K, Headey D, Singh A, Karmacharya C, Rana PP. Maternal and Child Nutrition in Nepal: Examining drivers of progress from the mid-1990s to 2010s. Glob Food Secur-AgricPolicy. 2017;13:30-7.*

da Silva ICM, Franca GV, Barros AJD, Amouzou A, Krasevec J, Victora CG. Socioeconomic Inequalities Persist Despite Declining Stunting Prevalence in Low- and Middle-Income Countries. J Nutr. 2018;148(2):254-8.

Darrouzet-Nardi AF. Nonviolent civil insecurity is negatively associated with subsequent height-for-age in children aged <5 y born between 1998 and 2014 in rural areas of Africa. Am J Clin Nutr. 2017;105(2):485-93.

Das Gupta ML, Michael; Gragnolati, Michele; Ivaschenko, Oleksiy. Improving Child Nutrition Outcomes in India: Can the Integrated Child Development Services Program Be More Effective? Washington DC; 2005. Contract No.: No. 3647.

Doctor HV, Nkhana-Salimu S. Trends and Determinants of Child Growth Indicators in Malawi and Implications for the Sustainable Development Goals. AIMS Public Health. 2017;4(6):590-614.

Efevbera Y, Bhabha J, Farmer PE, Fink G. Girl child marriage as a risk factor for early childhood development and stunting. Social science & medicine. 2017;185:91-101.

Ervin PA, Bubak V. Closing the rural-urban gap in child malnutrition: Evidence from Paraguay, 1997-2012. Econ Hum Biol. 2019;32:1-10.*

Goyal N, Canning D. Exposure to Ambient Fine Particulate Air Pollution in Utero as a Risk Factor for Child Stunting in Bangladesh. Int J Environ Res Public Health. 2017;15(1):23.

Greffeuille V, Sophonneary P, Laillou A, Gauthier L, Hong R, Hong R, et al. Persistent Inequalities in Child Undernutrition in Cambodia from 2000 until Today. Nutrients. 2016;8(5):16.

Hangoma P, Aakvik A, Robberstad B. Explaining changes in child health inequality in the run up to the 2015 Millennium Development Goals (MDGs): The case of Zambia. PLoS One. 2017;12(2):e0170995.

Harttgen K, Klasen S, Vollmer S. Economic Growth and Child Undernutrition in sub-Saharan Africa. Population and Development Review. 2013;39(3):397-412.

Hasan MT, Soares Magalhaes RJ, Williams GM, Mamun AA. The role of maternal education in the 15-year trajectory of malnutrition in children under 5 years of age in Bangladesh. Matern Child Nutr. 2016;12(4):929-39.

Headey D. An analysis of trends and determinants of child undernutrition in Ethiopia, 2000-2011. International Food Policy Research Institute (IFPRI); 2014. Contract No.: 70.*

Headey D, Hirvonen K, Hoddinott J. Animal Sourced Foods and Child Stunting. American Journal of Agricultural Economics. 2018;100(5):1302-19.

Headey D, Hoddinott J, Ali D, Tesfaye R, Dereje M. The Other Asian Enigma: Explaining the Rapid Reduction of Undernutrition in Bangladesh. World Development. 2015;66:749-61.*

Headey D, Hoddinott J, Park S. Drivers of nutritional change in four South Asian countries: a dynamic observational analysis. Matern Child Nutr. 2016;12 Suppl 1:210-8.*

Headey D, Hoddinott J, Park S. Accounting for nutritional changes in six success stories: A regression-decomposition approach. Glob Food Secur-AgricPolicy. 2017;13:12-20.*

Headey DD, Hoddinott J. Understanding the Rapid Reduction of Undernutrition in Nepal, 2001-2011. PLoS One. 2015;10(12):e0145738.*

Hoffman D, Cacciola T, Barrios P, Simon J. Temporal changes and determinants of childhood nutritional status in Kenya and Zambia. J Health Popul Nutr. 2017;36(1):27.

Huda TM, Hayes A, El Arifeen S, Dibley MJ. Social determinants of inequalities in child undernutrition in Bangladesh: A decomposition analysis. Matern Child Nutr. 2018;14(1).

Huicho L, Huayanay-Espinoza CA, Herrera-Perez E, Segura ER, Nino de Guzman J, Rivera-Ch M, et al. Factors behind the success story of under-five stunting in Peru: a district ecological multilevel analysis. BMC Pediatr. 2017;17(1):29.

Ikeda N, Irie Y, Shibuya K. Determinants of reduced child stunting in Cambodia: analysis of pooled data from three demographic and health surveys. Bull World Health Organ. 2013;91(5):341-9.*

Jahagirdar D, Harper S, Heymann J, Swaminathan H, Mukherji A, Nandi A. The effect of paid maternity leave on early childhood growth in low-income and middle-income countries. BMJ Glob Health. 2017;2(3):e000294.

Joe W, Rajaram R, Subramanian SV. Understanding the null-to-small association between increased macroeconomic growth and reducing child undernutrition in India: role of development expenditures and poverty alleviation. Matern Child Nutr. 2016;12 Suppl 1:196-209.

Joshi N, Bolorhon B, Narula I, Zhu S, Manaseki-Holland S. Social and environmental determinants of child health in Mongolia across years of rapid economic growth: 2000-2010. Int J Equity Health. 2017;16(1):189.

Kabubo-Mariara J, Ndenge GK, Mwabu DK. Determinants of Children's Nutritional Status in Kenya: Evidence from Demographic and Health Surveys. Journal of African Economies. 2009;18(3):363-87.

Khatun W, Rasheed S, Alam A, Huda TM, Dibley MJ. Assessing the Intergenerational Linkage between Short Maternal Stature and Under-Five Stunting and Wasting in Bangladesh. Nutrients. 2019;11(8):07.

Kien VD, Lee HY, Nam YS, Oh J, Giang KB, Van Minh H. Trends in socioeconomic inequalities in child malnutrition in Vietnam: findings from the Multiple Indicator Cluster Surveys, 2000-2011. Glob Health Action. 2016;9:29263.

Kinyoki DK, Berkley JA, Moloney GM, Kandala NB, Noor AM. Predictors of the risk of malnutrition among children under the age of 5 years in Somalia. Public health nutrition. 2015;18(17):3125-33.

Kov P, Smets , S., Spears, D., Vyas, S. Growing taller among toilets : evidence from changes in sanitation and child height in Cambodia, 2005-2010. Amston; 2013.

Krishna A, Mejia-Guevara I, McGovern M, Aguayo VM, Subramanian SV. Trends in inequalities in child stunting in South Asia. Matern Child Nutr. 2018;14 Suppl 4:e12517.

Lovo S, Veronesi M. Crop Diversification and Child Health: Empirical Evidence From Tanzania. Ecological Economics. 2019;158:168-79.

Masibo PK, Makoka D. Trends and determinants of undernutrition among young Kenyan children: Kenya Demographic and Health Survey; 1993, 1998, 2003 and 2008-2009. Public health nutrition. 2012;15(9):1715-27.

Matanda DJ, Mittelmark MB, Kigaru DM. Child undernutrition in Kenya: trend analyses from 1993 to 2008-09. BMC Pediatr. 2014;14:5.

Milman A, Frongillo EA, de Onis M, Hwang JY. Differential improvement among countries in child stunting is associated with long-term development and specific interventions. J Nutr. 2005;135(6):1415-22.

Mohsena M, Goto R, Mascie-Taylor CG. Socioeconomic and Demographic Variation in Nutritional Status of under-Five Bangladeshi Children and Trend over the Twelve-Year Period 1996-2007. J Biosoc Sci. 2017;49(2):222-38.

Monteiro CA, Benicio MH, Conde WL, Konno S, Lovadino AL, Barros AJ, et al. Narrowing socioeconomic inequality in child stunting: the Brazilian experience, 1974-2007. Bull World Health Organ. 2010;88(4):305-11.

Monteiro CA, Benicio MH, Konno SC, Silva AC, Lima AL, Conde WL. Causes for the decline in child under-nutrition in Brazil, 1996-2007. Rev Saude Publica. 2009;43(1):35-43.

Nepali S, Simkhada P, Davies I. Trends and inequalities in stunting in Nepal: a secondary data analysis of four Nepal demographic health surveys from 2001 to 2016. BMC Nutrition. 2019;5(1).

Nie P, Rammohan A, Gwozdz W, Sousa-Poza A. Changes in Child Nutrition in India: A Decomposition Approach. Int J Environ Res Public Health. 2019;16(10):22.

Nisar YB, Dibley MJ, Aguayo VM. Iron-Folic Acid Supplementation During Pregnancy Reduces the Risk of Stunting in Children Less Than 2 Years of Age: A Retrospective Cohort Study from Nepal. Nutrients. 2016;8(2):67.

Ntenda PAM, Chuang YC. Analysis of individual-level and community-level effects on childhood undernutrition in Malawi. Pediatr neonatol. 2018;59(4):380-9.

O'Donnell O, Nicolas AL, Van Doorslaer E. Growing richer and taller: Explaining change in the distribution of child nutritional status during Vietnam's economic boom. Journal of Development Economics. 2009;88(1):45-58.

Otterbach S, Rogan M. Exploring spatial differences in the risk of child stunting: Evidence from a South African national panel survey. Journal of Rural Studies. 2019;65:65-78.

Pierce H. Increasing health facility deliveries in Cambodia and its influence on child health. Int J Equity Health. 2019;18(1):67.

Ponce N, Shimkhada R, Raub A, Daoud A, Nandi A, Richter L, et al. The association of minimum wage change on child nutritional status in LMICs: A quasi-experimental multi-country study. Glob Public Health. 2018;13(9):1307-21.

Rabassa M, Skoufias, E., Jacoby, H. Weather and child health in rural Nigeria.: World Bank 2012. Report No.: 1813-9450 Contract No.: No. 6214.

Rabbani A, Khan A, Yusuf S, Adams A. Trends and determinants of inequities in childhood stunting in Bangladesh from 1996/7 to 2014. Int J Equity Health. 2016;15(1):186.

Rannan-Eliya RP, Hossain SM, Anuranga C, Wickramasinghe R, Jayatissa R, Abeykoon AT. Trends and determinants of childhood stunting and underweight in Sri Lanka. Ceylon Med J. 2013;58(1):10-8.

Rashad A, Sharaf M. Does economic growth reduce child malnutrition in Egypt? New evidence from national demographic and health survey. Working Papers - Department of Economics, University of Alberta; 2015 (2015-16):26 pp 25 ref. Alberta: University of Alberta; 2015.

Restrepo-Mendez MC, Barros AJ, Black RE, Victora CG. Time trends in socio-economic inequalities in stunting prevalence: analyses of repeated national surveys. Public health nutrition. 2015;18(12):2097-104.

Rieger M, Trommlerova SK, Ban R, Jeffers K, Hutmacher M. Temporal stability of child growth associations in Demographic and Health Surveys in 25 countries. SSM Popul Health. 2019;7:100352.

Saha UR, Chattapadhayay A, Richardus JH. Trends, prevalence and determinants of childhood chronic undernutrition in regional divisions of Bangladesh: Evidence from demographic health surveys, 2011 and 2014. PLoS One. 2019;14(8):e0220062.

Smith T, Shively G. Multilevel analysis of individual, household, and community factors influencing child growth in Nepal. BMC Pediatr. 2019;19(1):91.

Sobrino M, Gutierrez C, Alarcon J, Davila M, Cunha AJ. Birth interval and stunting in children under five years of age in Peru (1996-2014). Child Care Health Dev. 2017;43(1):97-103.

Spears D. How much international variation in child height can sanitation explain ? . Washington, DC.: World Bank Group; 2013. Contract No.: no. WPS 6351.

Stifel DA, H. . The “Glass of Milk” Subsidy Program and Malnutrition in Peru. The World Bank Economic Review. 2006;20(3):421-48.

Subramanyam MA, Kawachi I, Berkman LF, Subramanian SV. Socioeconomic inequalities in childhood undernutrition in India: analyzing trends between 1992 and 2005. PLoS One. 2010;5(6):e11392.

Subramanyam MA, Kawachi I, Berkman LF, Subramanian SV. Is economic growth associated with reduction in child undernutrition in India? PLoS medicine. 2011;8(3):e1000424.

Sunil TS, Sagna M. Decomposition of childhood malnutrition in Cambodia. Matern Child Nutr. 2015;11(4):973-86.

Surkan PJ, Ettinger AK, Ahmed S, Minkovitz CS, Strobino D. Impact of maternal depressive symptoms on growth of preschool- and school-aged children. Pediatrics. 2012;130(4):e847-55.

Urke HB, Mittelmark MB, Valdivia M. Trends in stunting and overweight in Peruvian pre-schoolers from 1991 to 2011: findings from the Demographic and Health Surveys. Public health nutrition. 2014;17(11):2407-18.

Wagstaff A, van Doorslaer E, Watanabe N. On decomposing the causes of health sector inequalities with an application to malnutrition inequalities in Vietnam. Journal of Econometrics. 2003;112(1):207-23.

Woodruff BA, Wirth JP, Bailes A, Matji J, Timmer A, Rohner F. Determinants of stunting reduction in Ethiopia 2000 - 2011. Matern Child Nutr. 2017;13(2).

Woodruff BA, Wirth JP, Ngnie-Teta I, Beauliere JM, Mamady D, Ayoya MA, et al. Determinants of Stunting, Wasting, and Anemia in Guinean Preschool-Age Children: An Analysis of DHS Data From 1999, 2005, and 2012. Food Nutr Bull. 2018;39(1):39-53.

World Bank. Maintaining Momentum to 2015 : An Impact Evaluation of Interventions to Improve Maternal and Child Health and Nutrition in Bangladesh. Washington, DC. : World Bank, Operations Evaluation Department; 2005.*

World Bank. Stunting reduction in Sub-Saharan Africa Washington, D.C.: World Bank Group; 2017.

Wu L, Yang Z, Yin SA, Zhu M, Gao H. The relationship between socioeconomic development and malnutrition in children younger than 5 years in China during the period 1990 to 2010. Asia Pac J Clin Nutr. 2015;24(4):665-73.

Yang YY, Kaddu G, Ngendahimana D, Barkoukis H, Freedman D, Lubaale YA, et al. Trends and determinants of stunting among under-5s: evidence from the 1995, 2001, 2006 and 2011 Uganda Demographic and Health Surveys. Public health nutrition. 2018;21(16):2915-28.

Zanello G, Srinivasan CS, Shankar B. What Explains Cambodia's Success in Reducing Child Stunting-2000-2014? PLoS One. 2016;11(9):e0162668.

Supplementary Table 2. Summary of stunting determinants and covariates assessed in analyses using national-level survey data.

Domain	Subgroupings
**IMMEDIATE CAUSES (PROXIMAL)**	
**Fertility**	• Total fertility rate
**Maternal Characteristics & Exposures during Pregnancy**	• Maternal height<br>• Maternal underweight /BMI<br>• Maternal malaria/anaemia<br>○ Iron supplementation during pregnancy<br>• Maternal age<br>○ Child marriage<br>• Mother's marital status<br>• Maternal physical and mental health<br>○ maternal HIV status<br>○ Maternal tobacco<br>○ PM2.5 exposure in utero<br>○ Chronic energy deficiency<br>• Maternal vaccination<br>○ Tetanus toxoid vaccine during pregnancy<br>• Parity<br>• Birth order<br>• Inter-pregnancy interval
**Dietary Intake**	• Food intake<br>• Micronutrient supplementation<br>• Protein supplements<br>• Infant and young child feeding (IYCF) practices<br>• Breastfeeding<br>• Complementary feeding<br>• Dietary diversity score
**Child Morbidities & Infectious Diseases**	• Infectious comorbidities (diarrhea, pneumonia, cough, fever)<br>• Micronutrient deficiencies (zinc, iron)<br>• WHZ/WAZ for older children
**Birth Outcomes**	• Preterm birth<br>• Small for gestational age birth<br>• Low birthweight<br>• Birth complication<br>• Birth weight<br>• Type of birth (singleton or multiple)<br>• WAZ/WHZ by gestational age
**UNDERLYING CAUSES (INTERMEDIATE II)**	
**Food Security**	• Access to food<br>• Food consumption per person (CNY)
**Health Services**	• Antenatal care coverage<br>• Skilled birth attendance coverage<br>• Visits to doctor/other health professionals<br>• Vaccination coverage<br>• Individual vaccination status<br>• Distance to health facility<br>• Place of birth<br>• Skilled delivery<br>• Composite Coverage Index (CCI)<br>○ Health service index<br>• Access to “Health care”<br>• Number of health facilities<br>• Contraceptive use
**Household Environment**	• Water, Sanitation, and Hygiene (WASH)<br>○ Access to clean water<br>○ Open defecation<br>○ Improved Toilets

	○ Piped Water<br>○ Sanitation infrastructure<br>● Urbanization (proportion living in urban areas)<br>● Household size/crowding<br>● Cooking with fuel<br>● Indoor and outdoor air pollution
**BASIC DETERMINANTS (INTERMEDIATE I)**	
**Other Programs, Policies & Interventions**	● Cash transfer program coverage<br>● Comprehensive health insurance (SIS) utilization<br>● Legislated paid maternity leave<br>● National nutrition program
**BASIC DETERMINANTS (DISTAL)**	
**Literacy**	● Maternal education<br>● Adolescent literacy<br>● Paternal education<br>● Population literacy rates<br>● Parental schooling
**Household Socioeconomic Status**	● Wealth quintiles/quartiles<br>● ABEP<br>● Asset/wealth index/ possession index/household income<br>○ Housing type<br>○ access to internet<br>○ Electricity<br>○ Possession score<br>▪ Livestock<br>● Poverty rate<br>● Occupation<br>● Caste<br>● Dependency ratio<br>● Household spending<br>○ Engel Coefficient<br>○ Household consumption per capita
**Women's Empowerment**	● Female headed households<br>● Can walk to health clinic alone<br>● Decision-making
**Macro level: Environmental & Community Context**	● Rainfall<br>● Seasonality<br>● Monsoon<br>● Drought<br>● Temperature<br>● Environmental Vulnerability Index (EVI)<br>● Community HIV/health status<br>○ Male to female life expectancy ratio<br>● Blood lead, for toxicity<br>● Village proportions<br>○ Households with bed nets,<br>○ Village population density<br>○ Households with electricity<br>○ Village proportion of open defecation<br>● Community wealth<br>● Female education in community
**Macro level: Economic Context**	● GDP<br>○ per capita net state domestic product<br>○ Income group<br>○ Economic growth<br>● Gini coefficient<br>● Poverty rate<br>○ Poverty head count ratio<br>○ Proportion with ≥1 unmet basic needs<br>● National policies<br>● Infrastructure<br>○ mobile phone penetration<br>○ per capita state developmental expenditure

	• log per capita health expenditures, public (PPP)<br>• population level personal/house income and spending<br>○ % share of working population who are wage and salary workers<br>○ Minimum wage change over time<br>○ Per capita income<br>○ Average Disposable Income per Capita<br>○ Average Consumption Expenditure per capita<br>○ ECRSC: Education, Cultural and Recreation Services Consumption per person.<br>• Food prices
**Macro level: Conflict & Insecurity**	• Corruption<br>• Conflict<br>○ Political violence
**Macro level: Political Context**	• Democracy<br>• Government effectiveness<br>• Political stability<br>• Rule of law<br>• State fragility index<br>• Regulatory quality
**OTHER**	
Miscellaneous	• Paternal height<br>• Mother dead or absent<br>• Father dead or absent
Key categories for stratification	• Gender<br>• Age<br>• Siblings<br>• Ethnicity<br>• Religion<br>• Region<br>• Geography<br>• Urban vs. rural<br>• Age/sex of household head<br>• Indigenous status<br>• Year

Supplementary Table 4. Summary of changes in stunting inequality predicted by changes in stunting determinant indicators.

			Bangladesh			Bolivia	Brazil	Columbia	Dominican Republic	Haiti	Honduras	India	Nepal		Nigeria		Peru	Vietnam			Zambia
			Huda 2018		Rabbani 2016	Amarante 2018	Amarante 2018	Amarante 2018	Amarante 2018	Amarante 2018	Amarante 2018	Nie 2019	Angdembe 2019		Akombi 2018		Amarante 2018	Kien 2016	O'Donnell 2009 ***	Wagstaff 2003 ***	Hangoma 2017
	Category	Determinant/Indicator	2004	2014	1996-2014	2003-2008	1996-2006	2005-2010	2002-2007	2006-2012	2005-2011	2004-2012	1996	2016	2003	2013	2007-2012	2000-2011	1993-1998	1993-1998	2007-2014
Basic Causes	Household SES	Asset Index/ Wealth Index			26%	-27%	154%	58%	588%	176%	47%	21%			186%	264%	113%				
		Wealth Quantile	18%	23%									61%	72%				64%			7%
		Household Consumption																	25%	74%	
		Mother Currently Employed	1%	3%											61%	106%					9%
	Literacy	Maternal Education	17%	26%	17%	19%	28%	13%	-13%	33%	126%		12%	1%	71%	119%	-15%	2%		-11%	6%
		Paternal Education	5%	9%	-11%								6%	3%							
	Region	Rural Residence																3%			-30%
		Urban Residence				37%	-15%	7%	13%	-203%	-10%				-141%	-239%	-6%				
Underlying Causes	WASH	Safe Water (piped water, tubewell)				32%	-24%	7%	8%	214%	5%	4%					4%		6.00%	16%	6%
		Improved Sanitation	7%	3.0%			-52%	-11%	-97%	-92%	3%						1%			-5%	-23%
		Open Defecation											-12%	4%							
	Unhealthy Household Environment	Household Size																	30%		2%
		Number of Children Under 5				6.0%															-10%
	Health Services	Mother received antenatal care	14%	8%	3%								0%	2%							
		Place of Birth/ Delivered at Health Facility	5%	5%	11%								5%	2%							26%
	Feeding Practices	Breastfeeding			1%	2%	1%	5%	-3%	-62%	-1%		0%	0%			1%				13%
Immediate Causes	Maternal Characteristics	Parity				6%	26%	16%	-18%	31%	-25%						-2%				
		Birth Order	3%	0%	-5%								9%	9%							27%
		Interpregnancy Interval	5%	3%																	
		Maternal Weight/BMI				-4%	-11%	9%		86%	30%	1%	1%	12%			11%				12%
		Maternal Age	0%	0%		-3%	-11%	-2%	13%	26%	-16%				48%	71%	0%				1%
		Maternal Height	5%	5%	9%								0%	6%							25%
	Child Characteristics	Age			17%	-1%	2%	-2%	-5%	-46%	-14%		-1%	-2%	190%	352%	2%	22%			1%
		Sex																8%			2%
		Ethnicity/Religion				38%	1%						12%	-1%			-7%	-18%			
	Birth Outcomes	Size at Birth/Birthweight											6%	3%							3%
	Infectious comorbidities	Diarrhea				0.00%	8%	-3%	-47%	0.00%	-16%						-3%				
	Overall Variance (%) explained by model		75%	85%	41%	101%	102%	97%	438%	151%	120%	9%					98%	81%	50%	69%	76%

Footnotes:
***** = 0-10 years**
Amarante 2018: Mother's age ≤ age 17 at birth, child is indigenous
Nie 2019: Hygiene (water source; flushing toilet; handwashing), Mother's Characteristics (mother's BMI; mother's education)
Angdembe 2019: Maternal short stature (<145cm) & Child's age = 25-59 months
Akombi 2018: asset index, maternal education, maternal age all categorical, so value of group furthest away from reference group was taken

Asset index: poorest
Maternal education: no education
Maternal age: 15-24 years

N.B. The total variance is the variance calculated by the study authors. Some models have adjusted for other covariates that have not been included in this table.

Supplementary Figure 1. National Stunting Prevalence. Source: UNICEF/WHO/World Bank Joint Malnutrition Estimates (JME), 2019.

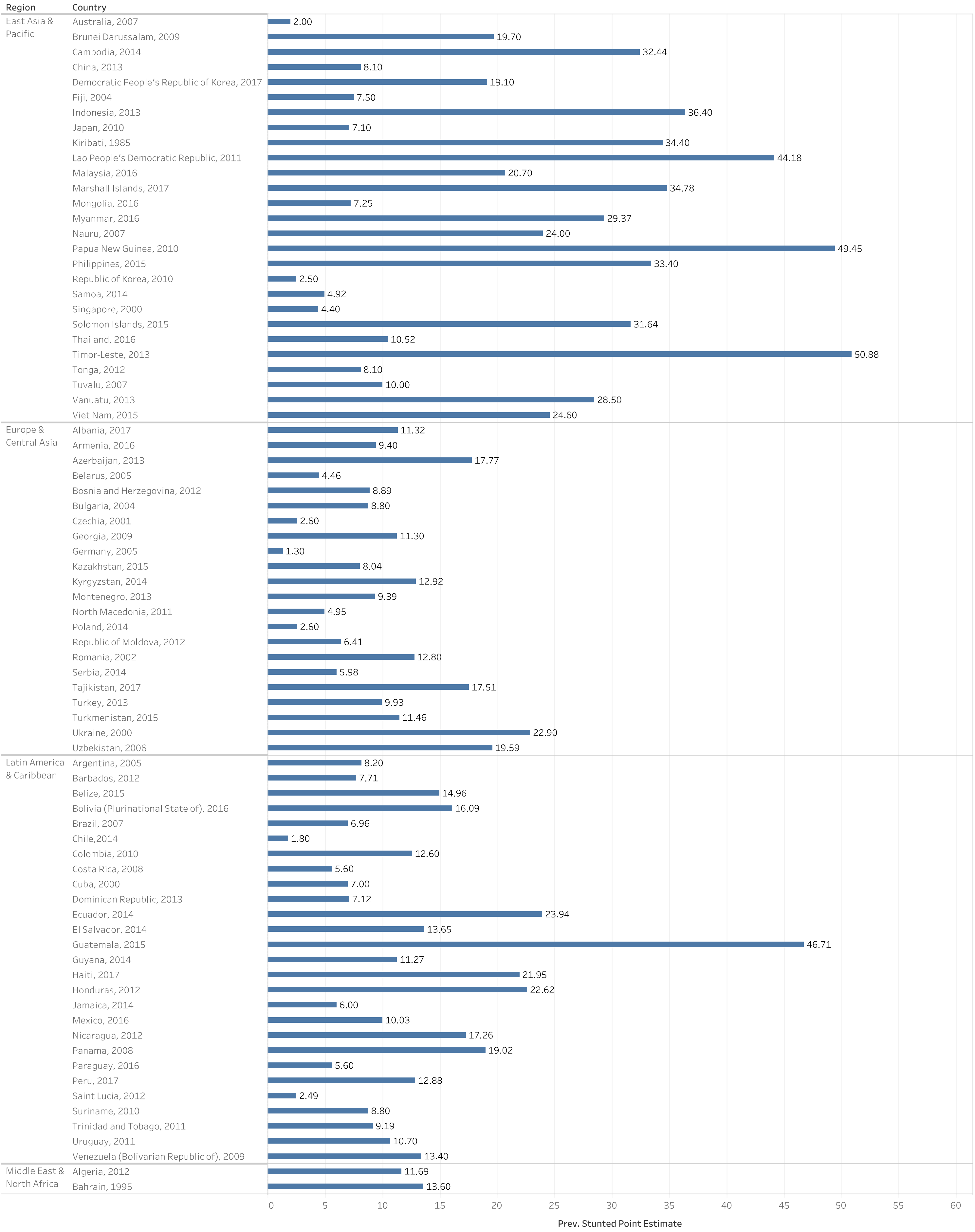

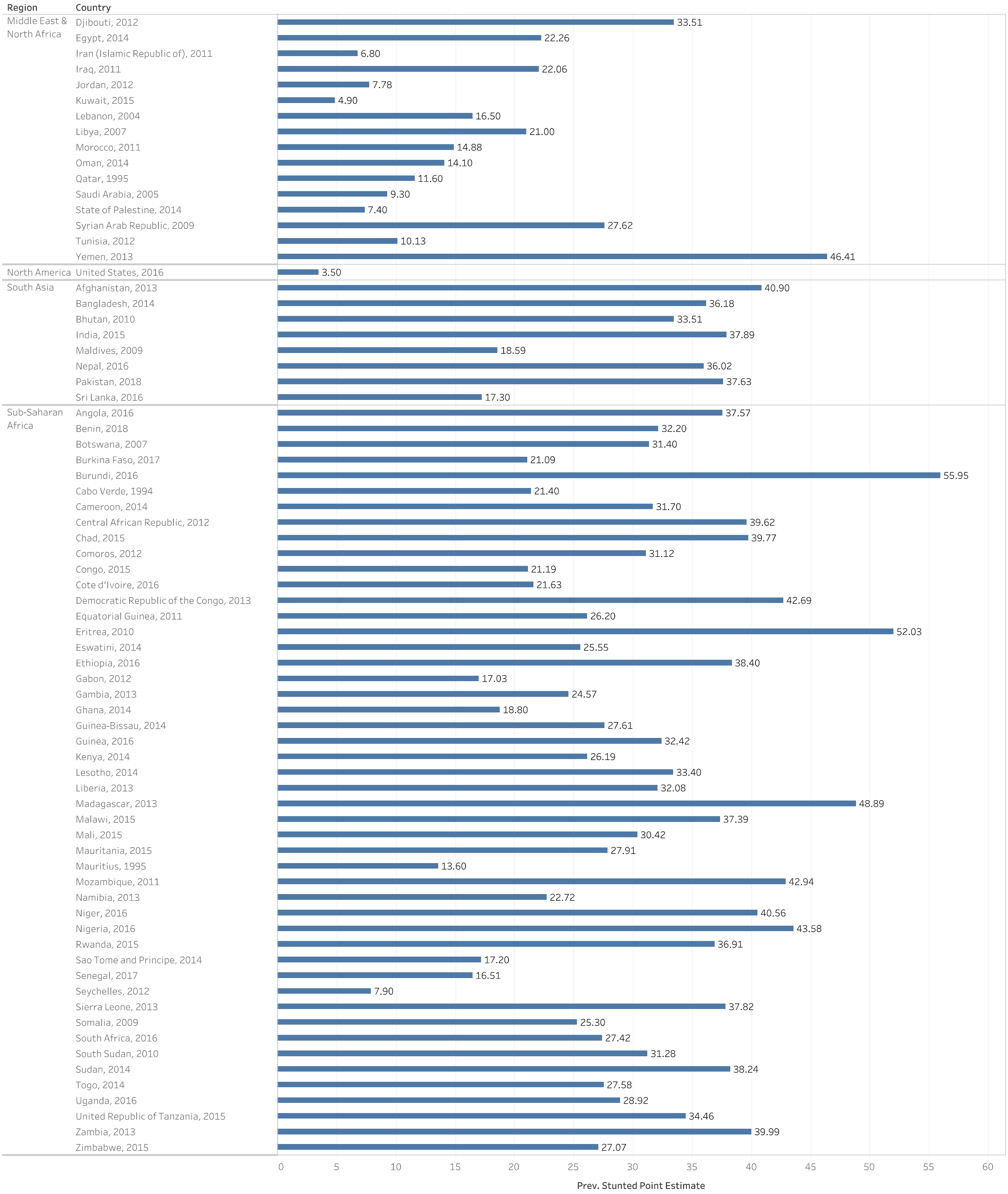

Sum of Prev. Stunted Point Estimate for each Country broken down by Region.

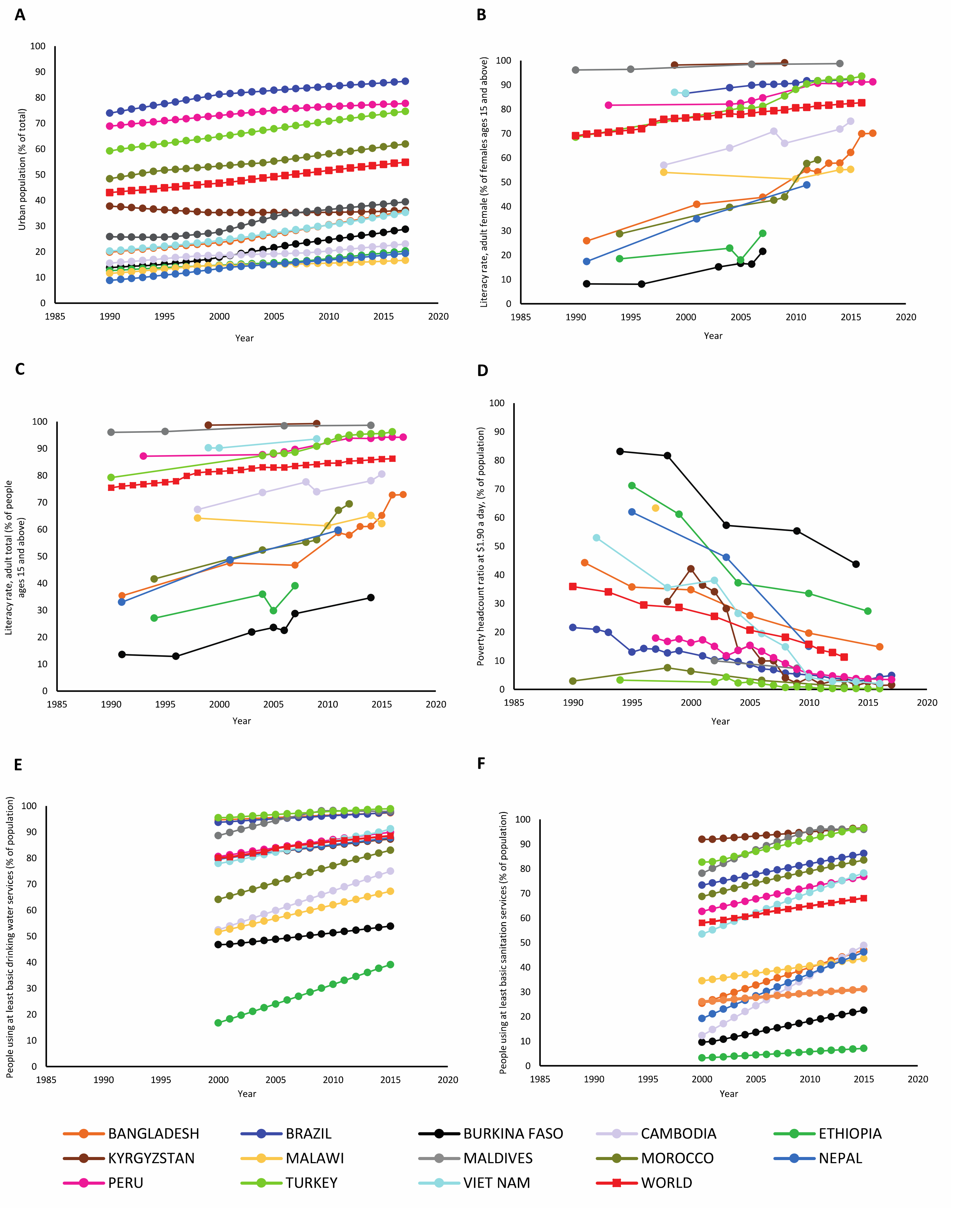

Supplementary Figure 2. Key indicator trends in top-performing countries: A, urban population; B, adult female literacy rate; C, adult literacy rate; D, poverty headcount ratio; E, access to basic drinking water services; F, access to basic sanitation services.

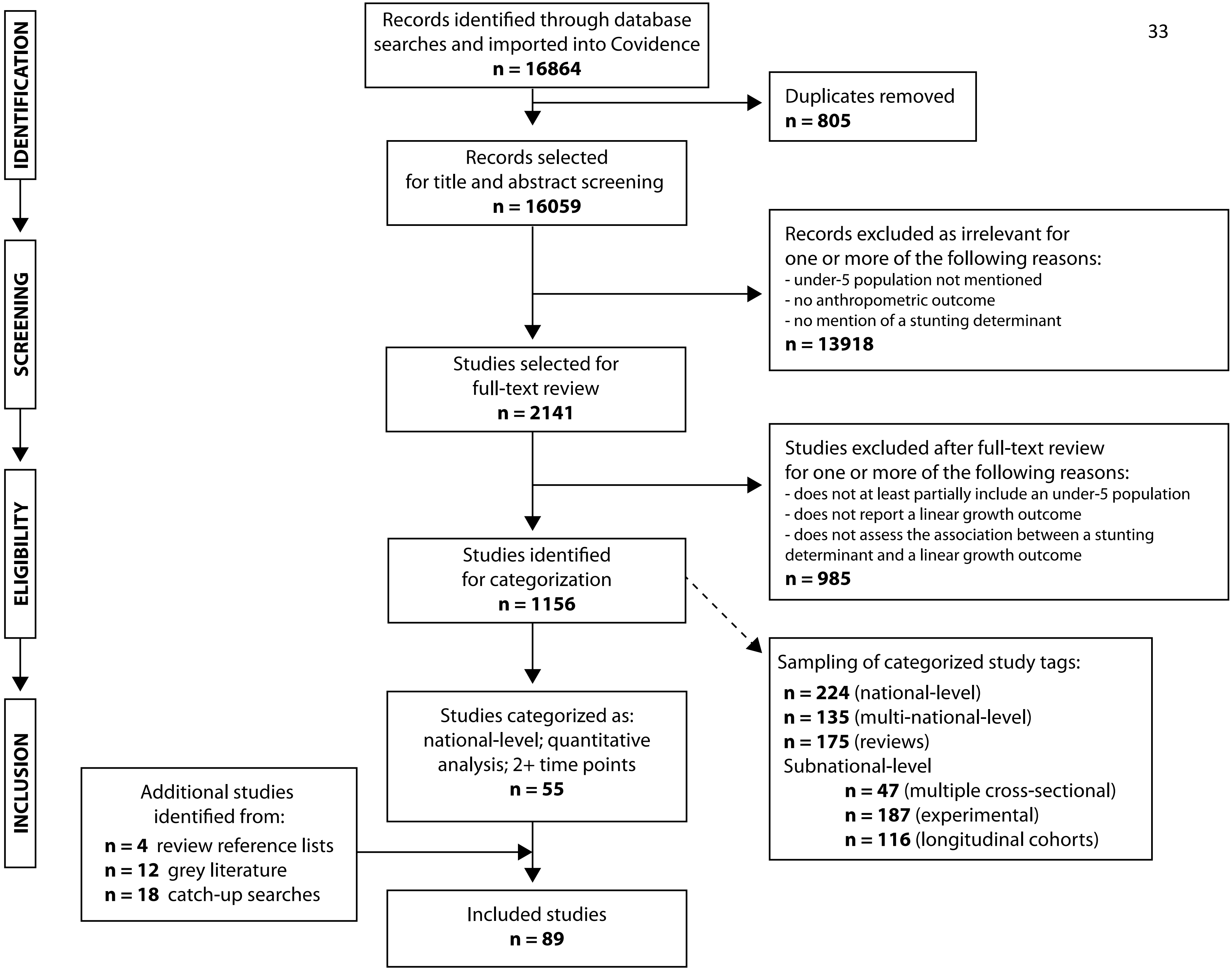

Supplementary Figure 3. Review flow diagram.
